# Integer-dimensional fractals of nonlinear dynamics, control mechanisms, and physical implications

**DOI:** 10.1038/s41598-018-28669-3

**Published:** 2018-07-09

**Authors:** Zonglu He

**Affiliations:** Faculty of Management and Economics Kaetsu, University 2-8-4 Minami-cho, Hanakoganei, Kodaira-shi, Tokyo 187-8578 Japan

## Abstract

Fractal dimensionality is accepted as a measure of complexity for systems that cannot be described by integer dimensions. However, fractal control mechanisms, physical implications, and relations to nonlinear dynamics have not yet been fully clarified. Herein we explore these issues in a spacetime using a nonlinear integrated model derived by applying Newton’s second law into self-regulating systems. We discover that (i) a stochastic stable fixed point exhibits self-similarity and long-term memory, while a deterministic stable fixed point usually only exhibits self-similarity, if our observation scale is large enough; (ii) stochastic/deterministic period cycles and chaos only exhibit long-term memory, but also self-similarity for even restorative delays; (iii) fractal level of a stable fixed point is controlled primarily by the wave indicators that reflect the relative strength of extrinsic to intrinsic forces: a larger absolute slope (smaller amplitude) indicator leads to higher positive dependence (self-similarity), and a relatively large amplitude indicator or an even restorative delay could make the dependence oscillate; and (iv) fractal levels of period cycles and chaos rely on the intrinsic resistance, restoration, and regulative delays. Our findings suggest that fractals of self-regulating systems can be measured by integer dimensions.

## Introduction

A typical statistical fractal^1^ exhibits statistical self-similarity^2–5^ with a power law^6^ and long-range dependence^7–12^. Fractal signals are ubiquitously observed in chemistry^13,14^, physics^15–17^, turbulence^18^, geology^19–21^, climate^6,22^, internet traffic^1,9^, hydrology^22,23^, biology^24–27^, medicine^4,28,29^, economics^30,31^, and city size distributions^32^. Fractal dimensions and their extensions such as multifractals^13,25,33^ provide statistical indexes to characterize fractals.

Fractal dimension as a measure of complexity does not uniquely determine patterns such as a strange attractor^34^; thus, different fractals have the same fractal dimension, and vice versa^35^. To more fully describe fractal patterns, fractal and multifractal dynamics of actual time series such as heartbeat and gait data have been modeled for the first time^36,37^. Stochastic fractal models have been proposed by incorporating fractal dimensions into models^38,39^, such as fractional Brownian motion, fractional Levy motion, obstructed diffusion, fractional Langevin equation, and autoregressive fractionally integrated moving average (ARFIMA) models. Methods developed by combining fractal parameters and fractal models have been used extensively to account for fractal properties such as power-law correlations in the variables^40,41^, coupling and decoupling of local and global self-affinity^16^, coupled fractal signals (ARFIMA–FIARCH)^42^, the spatiotemporal fractal cross-dependencies between coupled processes^43^, including new statistical methods such as efficient Gaussian maximum likelihood methods for vector ARFIMA^44^.

A fractal is thought to be the behavior of complex systems that cannot be described by an integer dimension. Asymmetric fractals can get very messy so fractals are regarded as the pictures of chaos. Some chaotic systems like the bifurcation diagram show fractal repetition. Chaos in fractal nonlinear systems and the effect of fractal order on chaotic control have been studied numerically^38^. But the relations of fractals to nonlinear dynamics and stochastic forcing have not been fully clarified^45^. Fractal-multifractal mechanisms of physiological variability in biological time-series outputs were proposed for the first time as a result of stochastic feedback among multiple dynamical elements that control given systems^36,37^. Imagining ubiquitous fractals without physics is difficult^46^. The physical mechanisms underlying fractal signals and the ubiquity of fractals need to be further explored.

This study adopted a completely different approach to solve these issues. Fractals are almost ubiquitous in the natural world which is a self-regulating system according to contemporary views of ecology and ecosystems. In a self-regulating system, there is a mechanism which tends to balance various influences and effects so that a stable behavior is maintained. Hence, a nonlinear autoregressive integrated (NLARI) model that characterizes a general self-regulating system^47^ was introduced in a multi-dimensional space-time. The aim is to reveal fractal signals of nonlinear dynamics from a stable fixed point to chaos in self-regulating systems and their control mechanisms and physical implications. Contrary to traditional methods used to incorporate fractal parameters into models, NLARI has its own fractal parameters.

A process in a self-regulating system can be generated by NLARI if the system sustains unpredictable disturbance ($\epsilon$), which may cause a deviation from the equilibrium or mean value; a general resistance force, which is a function of velocity, prevents a fast change due to perturbation and can be linearized for a low velocity; and a general restorative force (*g*), which is a function of the deviation from the mean $x-E(x)\equiv y$ that satisfies *yg*(*y*) < 0 for *y* ≠ 0 and *g*(*y*) = −*g*(−*y*) to reflect the nature of a restorative force that tends to pull the system back toward equilibrium, and it is absolutely integrable to avoid explosive solutions. By applying Newton’s second law to the system and discretizing it, NLARI can be derived and specified by

1 $$\begin{array}{rcl}{X}_{t} & = & {\theta }_{0}+(1+{\theta }_{1}){X}_{t-1}-{\theta }_{1}{X}_{t-2}+{\theta }_{2}\frac{-({X}_{t-{\kappa }_{2}}-{\mu }_{t-{\kappa }_{2}})}{\exp [{({X}_{t-{\kappa }_{2}}-{\mu }_{t-{\kappa }_{2}})}^{2}]}+{v}_{t}\\ {\theta }_{0} & = & \{\begin{array}{c}\frac{\omega }{1+\alpha }\\ \omega \end{array},\,{\theta }_{1}=\{\begin{array}{c}\frac{1}{1+\alpha }\\ 1-\alpha \end{array},\,{\theta }_{2}=\{\begin{array}{c}\frac{\beta }{1+\alpha }\\ \beta \end{array},\,{v}_{t}=\{\begin{array}{cc}\frac{{\varepsilon }_{t}}{1+\alpha } & {\rm{if}}\,{\kappa }_{1}=0\\ {\varepsilon }_{t} & {\rm{if}}\,{\kappa }_{1}=1\end{array}\end{array}$$

where ${\mu }_{t}\equiv E({X}_{t}|{X}_{0},\ldots ,{X}_{-{\kappa }_{2}})={X}_{0}+(\omega /\alpha )t$, $\omega =E({\epsilon }_{t})$, *ε*_*t*_ is Gaussian white noise with variance σ^**2**^; *α* is the resistance coefficient (0 < *α* < 2), *β* is the restoration coefficient (*β* > 0), and *κ*_1_ and *κ*_2_ are delays in the resistance and restoration (on its physical meaning see the Supplementary information). Let *Y*_*t*_ = *X*_*t*_ − *X*_0_ − (*ω*/*α*)*t*. Equation (1) can be written as

2 $${Y}_{t}=(1+{\theta }_{1}){Y}_{t-1}-{\theta }_{1}{Y}_{t-2}+{\theta }_{2}\frac{-\,{Y}_{t-{\kappa }_{2}}}{{e}^{{Y}_{t-{\kappa }_{2}}^{2}}}+{v}_{t}$$

When *v*_*t*_ = 0, equation (2) is the deterministic system that has exactly two solutions: a null fixed point ${y}_{1t}^{\ast }=0$ for null initial values and a two-period cycle ${y}_{2t}^{\ast }={(-1)}^{t}\sqrt{\mathrm{ln}\,\gamma }$ for non-null initial values in *κ*_1_ = 0, 1 and any odd number *κ*_2_. The relative restoration (or stability) coefficient *γ* = *β*/(4 ± 2*α*) controls its stability and bifurcation: the fixed point is exponentially asymptotically stable if 0 < *γ* < 1, the periodic cycle is exponentially asymptotically stable if $1 < \gamma  < \sqrt{e}$ but unstable if $\gamma  > \sqrt{e}$, and equation (1) with *γ* = 0 is nonstationary^47,48^. The related statistical properties have been developed^49^.

This study includes the following steps. The NLARI’ deterministic dynamics is incompletely clarified, thus we first derive a whole dynamic evolution in a 1 + 1-dimensional space-time. The second step is to reveal fractals of the deterministic/stochastic nonlinear dynamics and control parameters and to identify the impact of extrinsic disturbance on fractals. The third step is to clarify the relation between fractals and observation scales. The fourth step is to extend the (1 + 1)-dimensional space-time to a (1 + *m*)-dimensional space-time and discuss simple cases. Finally, we provide evidence for fractals.

## Results

### An evolutionary route of nonlinear dynamics

**Figure 1:**
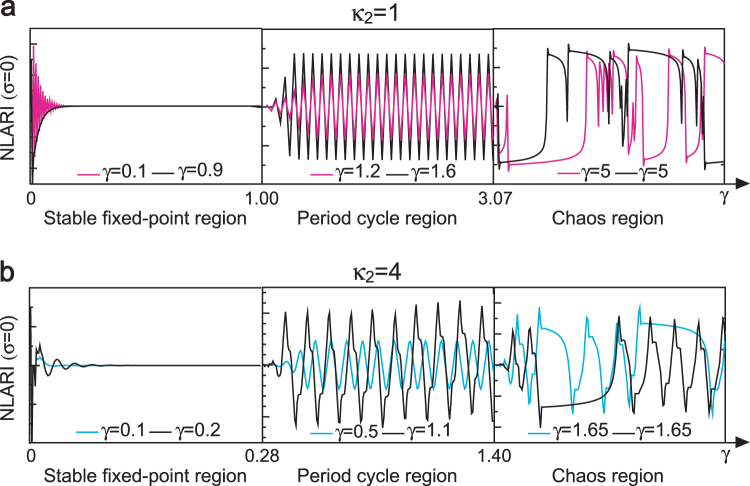
A whole evolutionary route of nonlinear dynamics. The stability coefficient *γ* controls the stability and dynamic patterns of the nonlinear autoregressive integrated (NLARI) process in the absence of noise. (**a**) For the resistant delay of *κ*_1_ = 1 and the restorative delay of *κ*_2_ = 1, the deterministic NLARI has a stable fixed point in 0 < *γ* < 1 (e.g., *γ* = 0.1 and *γ* = 0.9), a stable two-period cycle in $1\le \gamma  < \sqrt{e}$ (e.g., *γ* = 1.2 and *γ* = 1.6), an unstable two-period cycle in $\sqrt{e}\le \gamma  < 3.07$, and chaos in *γ* ≥ 3.07 (e.g., *γ* = 5 for different initial values). (**b**) For *κ*_1_ = 1 and *κ*_2_ = 4, the deterministic NLARI has a stable fixed point in 0 < *γ* < 0.28 (e.g., *γ* = 0.1 and *γ* = 0.2), multiple-period cycles in 0.28 ≤ *γ* < 1.4 (e.g., *γ* = 0.5 and *γ* = 1.1), and chaos in *γ* ≥ 1.4 (e.g., *γ* = 1.65 for different initial values).

Consider a (1 + 1)-dimensional space-time. Let ${\bf{W}}(t)=\{t,X(t)|t\in R\}$ be a continuous-time point and ${{\bf{W}}}_{t}=(t,{X}_{t}|t=1,2,\ldots )$ be a discrete-time point in the space-time. The trajectories of nonlinear dynamics in the space-time are a function of time; thus, their fractals are fractal signals. We demonstrated that as the stability coefficient increases, the deterministic NLARI with *κ*_1_ = 1 could undergo transitions from a stable to an unstable state and from a two-period to multiple-period cycle, finally moving towards chaos. The parity and the length of the restorative delays significantly affect nonlinear dynamics and speed up dynamic phase transitions. For example, the deterministic NLARI exhibits a stable fixed point in 0 < *γ* < 1, period cycles in 1 < *γ* < 3.07, and chaos in *γ* > 3.07 for one restorative delay (Fig. 1a), but is a stable fixed point in 0 < *γ* < 0.28, period cycles in 0.28 < *γ* < 1.4, and chaos in *γ* > 1.4 for four restorative delay (Fig. 1b). The next fractal study was performed along the evolutionary route of the nonlinear dynamics.

### Long-range dependence and control indicators

A process possesses long-range dependence if the quantity is equal to

3 $$\mathop{\mathrm{lim}}\limits_{N\to \infty }\,\sum _{n=-N}^{N}\,|{\rho }_{n}|=\infty $$

for the autocorrelation function (ACF) *ρ*_*n*_ at lag *n* (ref.^50^). If $|{\rho }_{n}|\le \frac{1}{n}$ for all *n*, then the process has long-range dependence or long-term memory, and the ACF of long-range dependence slowly decays at a hyperbolic rate or with an oscillation or other patterns as the lag increases. A slower (faster) decaying ACF indicates a higher (lower) level or degree of dependence.

To discover the dependence control indicators, we calculated the sample ACF using the data generated by equation (1). Simulation study indicated that the sample ACF could decay more slowly (the dependence could increase) with either a decreasing or increasing value of single NLARI parameters. Thus, any single NLARI parameter is inappropriate as a dependence control indicator. Consider the parameters ratios of *ω*/*α* and *σ*/*β*, which reflect the relative strength of extrinsic to intrinsic driving forces of the system, as candidate control indicators of fractals. Due to *E*(*X*_*t*_|*X*_0_, *X*_−1_) = *X*_0_ + (*ω*/*α*)*t*, *ω*/*α* is the slope of the mean line. Then, a wave slope indicator was defined by

4 $${\eta }_{1}=\frac{\omega }{\alpha }$$

We found that *ω*/*α* and *σ*/*β* were strongly positively correlated with the standard deviation (sd) of the data generated by equation (1) in the stable fixed-point, but the correlation became uncertainly in the period-cycle and chaos regions (see Supplementary information). This finding reveals the physical implication of the sd: the magnitude of the sd in the stable fixed point reflects the relative strength of the extrinsic to intrinsic driving forces of the system. The sd is a measure of how far the signal fluctuates from the mean; thus, a wave amplitude indicator was given by

5 $${\eta }_{2}=\frac{\sigma }{\beta }$$

**Figure 2:**
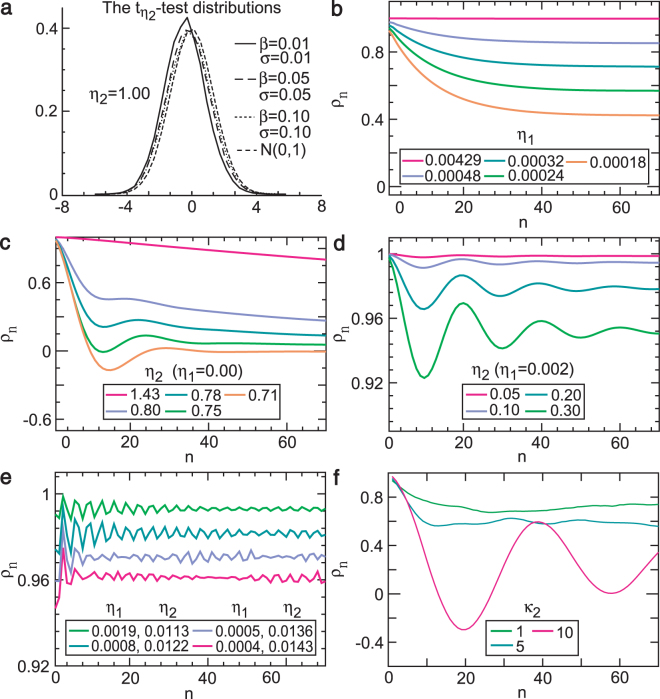
Long-range dependence of stochastic fixed point and control indicators. (**a**) The empirical distributions of the ${t}_{{\eta }_{{\bf{2}}}}$ statistic for the amplitude indicator *η*_2_ based on ordinary least squares estimates approximate a normal distribution for relatively small noise (e.g., *σ* ≤ 0.1). (**b**) As the slope indicator *η*_1_ increases from 0.00024 to 0.00429, the decay rate of the sample autocorrelation function (ACF) *ρ*_*n*_ against lag *n* decreases (the dependence increases). (**c**) When *η*_1_ = 0, the decay rate of the sample ACF reduces (the dependence increases) as the amplitude indicator increases from 0.75 to 1.43. (**d**) When *η*_1_ ≠ 0, the decay rate of the sample ACF increases (the dependence lowers) as the amplitude indicator increases from 0.05 to 0.30. (**e**) The decay rate of the sample ACF reduces (the dependence rises) as the slope indicator increases from 0.0004 to 0.0019 and the amplitude indicator decreases from 0.0143 to 0.0113, which supports cases (**b**–**d**). (**f**) Greatly increasing the restorative delay leads to long-range dependence with oscillations, which show the effect of the restorative delay on long-range dependence.

We demonstrated that the standard *t* statistic for testing the amplitude indicator has a limiting normal distribution under certain assumptions (see Methods). Empirical distributions of the *t*-*η*_2_ statistic that approximated the standard normal distribution for a relatively small variance (Fig. 2a) support this theoretical result. Thus, the common Student’s distribution can be used for testing the amplitude indicator if data satisfy those assumptions.

The wave indicators identified what controlled the dependence of NLARI in the stable fixed-point region (stochastic stable fixed point) with one restorative delay. We found that the absolute slope indicator primarily controls the dependence or dependence length of the stochastic stable fixed point in a positive relationship. For example, an increase of the absolute slope indicator lowers the decay of sample ACFs (the dependence increases) in different combinations of components (Fig. 2b). An increase of the amplitude indicator with *η*_1_ = 0 decelerates the decay of the ACFs (Fig. 2c), whereas an increase of the amplitude indicator with *η*_1_ ≠ 0 accelerates the decay rate of the ACFs (the dependence decreases) (Fig. 2d). As the absolute slope indicator increases and the amplitude indicator decreases, the decay of the ACFs slows up (Fig. 2e), which supports the result presented in Fig. 2c,d. A relatively large absolute slope indicator with a relatively large amplitude indicator (Fig. 2d,e) or an even restorative delay (Fig. 2f) could make the sample ACF oscillate.

**Figure 3:**
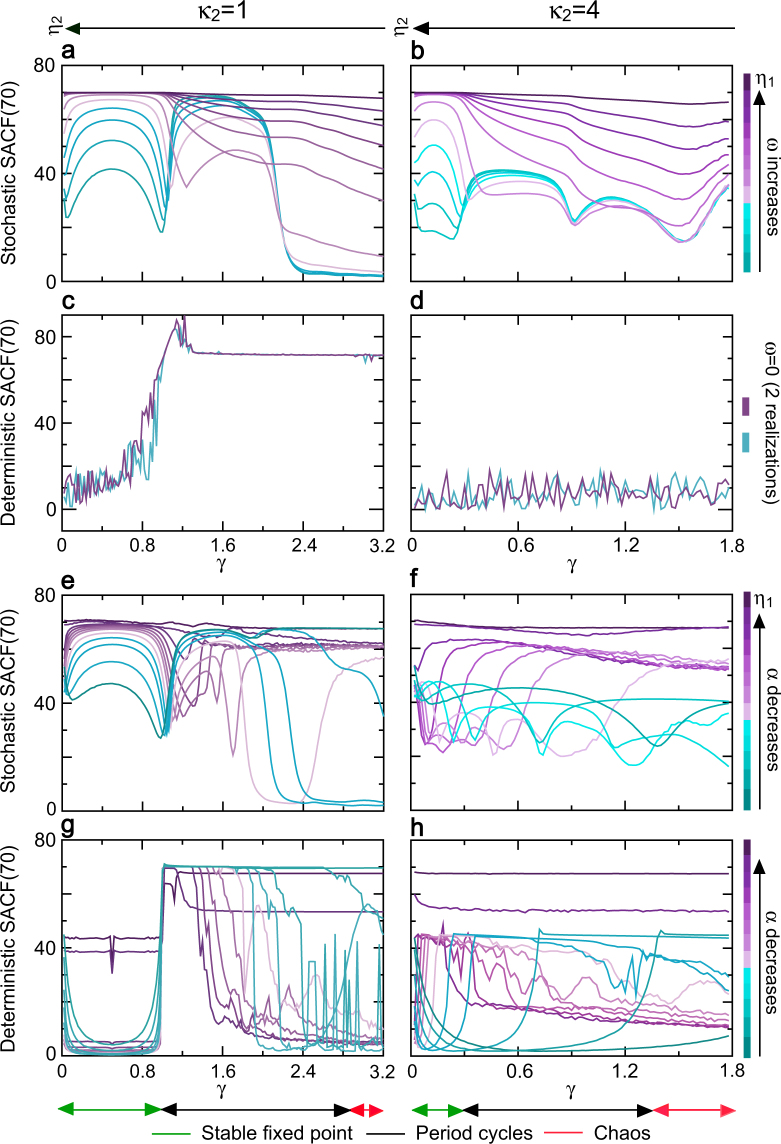
Long-range dependence of nonlinear dynamics and control mechanisms. SACF(70) denotes the partial sum of the first 70 terms of the simulated autocorrelation function sequence as a measure of dependence. A large SACF(70) reflects high level of dependence. (**a**,**b**) The traces of SACF(70) versus the stability coefficient *γ* for the NLARI’ stochastic stable fixed point, period cycles, and chaos when the slope indicator |*η*_1_| is increased by increasing the disturbance mean |*ω*| with the restorative delays of *κ*_2_ = 1 and *κ*_2_ = 4, respectively. (**c**,**d**) The traces of SACF(70) of the deterministic stable fixed point, period cycles, and chaos for the NLARI in (**a**,**b**) without noise for *κ*_2_ = 1 and *κ*_2_ = 4, respectively. (**e**,**f**) The traces of SACF(70) versus the stability coefficient *γ* for the NLARI’ stochastic stable fixed point, period cycles, and chaos when the slope indicator |*η*_1_| is increased by decreasing the resistance coefficient *α* for *κ*_2_ = 1 and *κ*_2_ = 4, respectively. (**g**,**h**) The traces of SACF(70) of the deterministic stable fixed point, period cycles, and chaos for the NLARI in (**e** and **f**) without noise for *κ*_2_ = 1 and *κ*_2_ = 4, respectively. In (**a**–**d**), *α* = 1.1 and the restoration coefficient $\beta \in (0.036,5.760)$. In (**e**–**h**), $\alpha \in (0.001,1.7)$ and $\beta \in (0.012,12.79)$. In (**a**,**b**,**e**,**f**), the value of the amplitude indicator *η*_2_ decreases as the value of the stability coefficient *γ* increases.

Then, we examined whether the dependence control function of the slope indicator was still valid in the whole dynamic region with odd/even restorative delays. Let SACF(N) denote the partial sum of the sample ACF sequence as a measure of the dependence for finite lag N. A larger SACF(N) indicates higher dependence level. To determine the effect of extrinsic disturbance on the dependence, SACF(70) was used in both deterministic and stochastic nonlinear dynamics of NLARI. As an example, Fig. 3 presents the traces of the simulation sample SACF(70) of the NLARI with and without noise versus the stability coefficient *γ* with one and four restorative delays. In noise case, each trace corresponds to the same slope indicator. For a given resistance coefficient (Fig. 3a–d), the NLARI’ stochastic stable fixed point, period cycles, and chaos can approach the maximum value of SACF(70) in which the SACF(N) increases as the absolute slope indicator |*η*_1_| increases (which is implemented by increasing the absolute disturbance mean |*ω*|). In contrast, the SACF(70) of the stochastic period cycles and chaos under a small absolute slope indicator can reach the maximum value and small values of SACF(70), respectively, whether the restorative delay is one (Fig. 3a) or four (Fig. 3b). The deterministic stable fixed point has small SACF(70), which is consistent with *η*_1_ = 0 due to the lack of noise; the deterministic period cycles and chaos with odd restorative delays reach the maximum value of SACF(70) (Fig. 3c); whereas all of the deterministic dynamics with even restorative delays have small SACF(70) (Fig. 3d). Similar fractal signals occur in a wide range of the resistance coefficient (Fig. 3e–h), but the dependence of period cycles and chaos become complex. We conclude that the absolute slope indicator controls a positive dependence of stochastic/deterministic stable fixed point; the stochastic stable fixed point can exhibit long-range dependence, while the deterministic stable fixed point has usually no long-range dependence; the deterministic/stochastic period cycles and chaos can have long-range dependence for odd restorative delays but can have no long-range dependence for even restorative delays; a small absolute slope indicator tends to increase the dependence of period cycles; and the amplitude indicator influences the dependence of nonlinear dynamics.

A relation between the slope indicator and the scaling exponent may be important to exactly measure fractal signals in the lack of a precise formulation of ACF. We estimated the value of the Hurst exponent by adopting rescaled range (R/S) analysis and detrended fluctuation analysis (DFA)^51^ with the data generated by the NLARI process used in Fig. 2b. As shown there, the degree of positive dependence in the stable fixed-point region rose as the slope indicator increased. The simulated correlation coefficients with 1000 alternations indicated a positive correlation with the 99.0% and 99.9% significance levels for the two methods, respectively. The increase of the estimated Hurst exponent corresponded to the increase of positive dependence, particularly the sample ACF plot in *H* = 0.90 was a horizontal line with almost no decay as the first trace in Fig. 2b (see Supplementary information).

### Self-similarity and control indicator

We say that a stochastic process is second-order self-similar if it satisfies

6 $${r}_{h}({X}_{m})={m}^{\delta }{r}_{h}(X)$$

where *r*_*h*_ is the lag-*h* autocovariance, *δ* ≥ 0, $X=({X}_{t}:t=1,\ldots ,T)$, *m* is any integer, and ${X}_{m}=({X}_{t}^{(m)}:t=1,$ $\ldots ,[T/m])$([*x*] means the integer part of *x*) is an aggregated point series over non-overlapping blocks of size *m*:

7 $${X}_{t}^{(m)}=\frac{1}{m}\,\sum _{k=1}^{m}\,{X}_{(t-1)m+k}$$

This definition is a discrete-time formulation of the definition introduced by Mandelbrot^52^. Rigorously speaking, the process defined by equation (6) is self-affine because *X*_*t*_ and *t* have to be scaled differently to make the function look similar^45^. But the performance of self-similarity based on the similarity ratios introduced below does not require different kinds of units. For this reason, the process defined in equation (6) is called self-similar process.

The autocovariance function of an actual time series is usually unknown. To characterize its self-similarity, we introduced the autocovariance similarity ratio based on the sample lag-*h* autocovariance function as a realistic and efficient measure. Equation (6) implies that *r*_*h*_(*X*) = *i*^−*δ*^*r*_*h*_(*X*_*i*_) and *r*_*h*_(*X*_*m*_) = *i*^−*δ*^*r*_*h*_(*X*_*im*_). We can write

8 $$\frac{{r}_{h}(X)}{{r}_{h}({X}_{m})}=\frac{{r}_{h}({X}_{2})}{{r}_{h}({X}_{2m})}=\cdots =\frac{{r}_{h}({X}_{i})}{{r}_{h}({X}_{im})}\equiv {r}_{h(i,im)}$$

or ${r}_{h(1,m)}={r}_{h(2,2m)}=\cdots ={r}_{h(i,im)}$. We call *r*_*h*(*i*,*im*)_ the lag-*h* autocovariance similarity ratio (or similarity ratio) and $s{d}_{(i,im)}=\sqrt{{r}_{0(i,im)}}$ the sd similarity ratio with the aggregation sizes *i* and *i* × *m* where *m* is called the similarity-ratio size. If the process is self-similar, then plotting the similarity ratio *r*_*h*(*i*,*im*)_ against size *i* is a roughly horizontal line; and the average similarity ratio ${r}_{hm}=\frac{1}{n}\,{\sum }_{i=1}^{n}\,{r}_{h(i,im)}$ obeys a power law *m*^−*δ*^ for any given *m*. If the two properties hold true, then the data support self-similarity.

**Figure 4:**
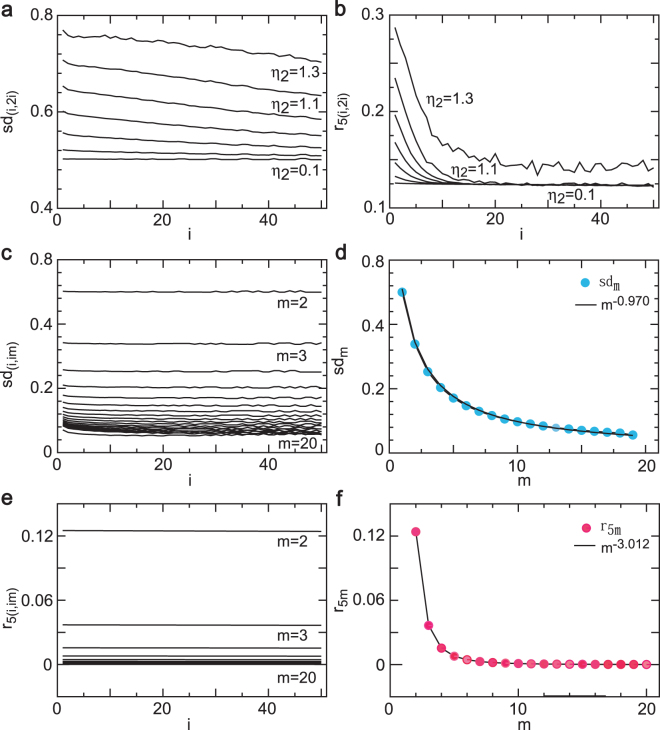
Self-similarity of stochastic fixed point and control indicator. (**a**,**b**) For *γ* = 0.286, as the amplitude indicator *η*_2_ decreases from 1.3 to 0.1, the trajectories of the simulated standard deviation similarity ratios with size 2 (*sd*_(*i*,**2***i*)_) and lag 5 similarity ratios with size 2 (*r*_**5**(*i*,**2***i*)_) change from descending lines to roughly horizontal lines, which shows that a relatively small amplitude indicator (*η*_**2**_) leads to self-similarity. (**c**) Plotting the simulated standard deviation similarity ratios (*sd*_(*i*,*im*)_) against size *i* generates roughly horizontal lines. (**d**) Average standard deviation similarity ratios (*sd*_*m*_) is a power function with power −0.970. (**e**) Simulated lag 5 autocovariance similarity ratios (*r*_**5**(*i*,*im*)_) versus size *i* results in perfectly horizontal lines. (**f**) Average autocovariance similarity ratio (*r*_**5***m*_) is a power function with power −3.012. In cases (**c**–**f**), the NLARI process possesses exact self-similarity for *γ* = 0.286 and *η*_**2**_ = 0.025. Consequently, the amplitude indicator controls the level of self-similarity: a smaller amplitude indicator corresponds to higher self-similarity in the stable fixed-point region.

The simulation similarity ratios and their average similarity ratio were used to determine self-similarity and control mechanism of the NLARI’ stochastic stable fixed point with one resistive delay and one restorative delay. As an example, Fig. 4 shows that the smaller the amplitude indicator, the higher level the self-similarity in the case. We see that the trace of the sd similarity ratios, *sd*_(*i*,**2***i*)_, versus size *i* changes from a descending line to a roughly horizontal line as the amplitude indicator decreases from 1.3 to 0.1 in the stable fixed-point region (e.g., *γ* = 0.286) (Fig. 4a). Similarly, the plot of the lag 5 similarity ratios, *r*_**5**(*i*,**2***i*)_, versus size *i* exhibits a roughly horizontal line under the amplitude indicator of *η*_2_ = 0.1 (Fig. 4b). Furthermore, our simulation results confirm that a small amplitude indicator (e.g., *η*_2_ = 0.025) can lead to self-similarity for *γ* = 0.286: plotting the sd similarity ratio against size yields a roughly horizontal line (Fig. 4c) and the average similarity ratio is a power function with power −0.970 (Fig. 4d). The lag 5 similarity ratio against size also displays an almost horizontal line (Fig. 4e) and the average similarity ratio is a power function with power −3.012 (Fig. 4f). These results indicate that the amplitude indicator controls the level of self-similarity of the stable fixed point with an odd restorative delay and that the power index can be larger than one.

**Figure 5:**
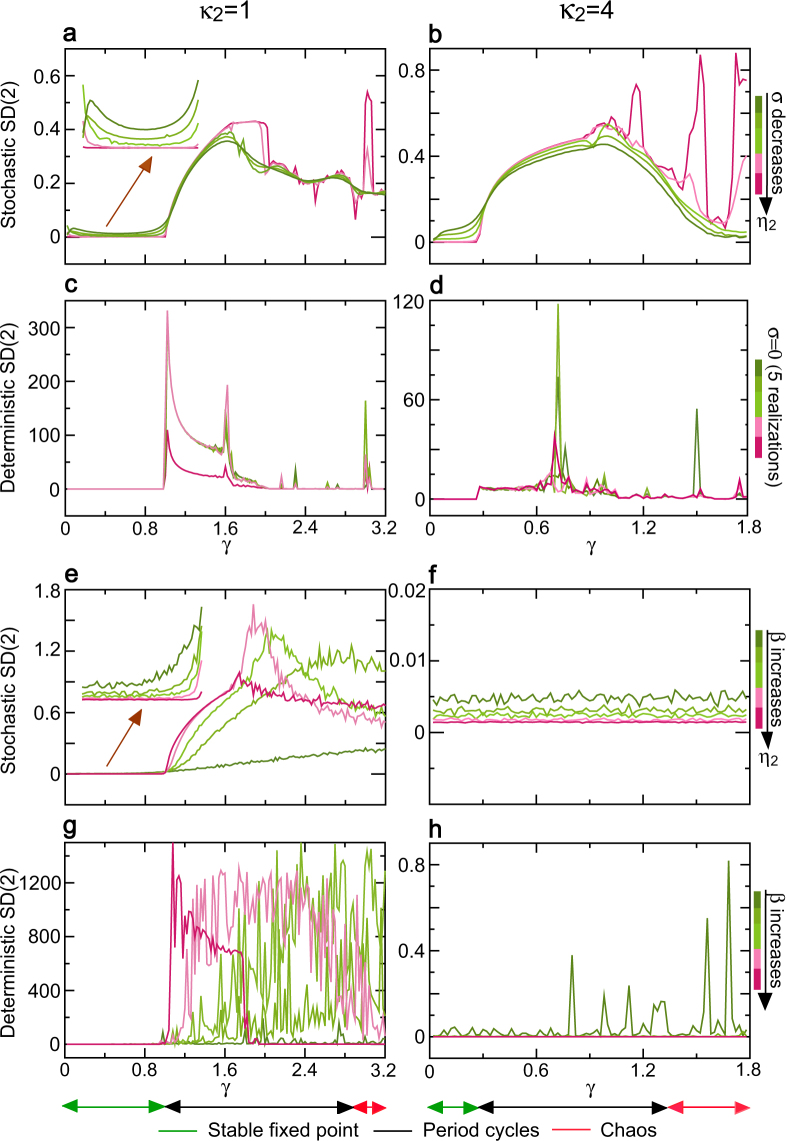
Self-similarity of nonlinear dynamics and control mechanisms. SD(2) denotes the standard deviation of the similarity ratio sequence with size two as a measure of self-similarity level. A near zero SD(2) reflects high level of self-similarity. (**a**,**b**) The traces of SD(2) versus the stability coefficient *γ* for the NLARI’ stochastic stable fixed point, period cycles, and chaos when the amplitude indicator *η*_**2**_ is decreased by decreasing the disturbance standard deviation *σ* with the restorative delays of *κ*_**2**_ = 1 and *κ*_**2**_ = 4, respectively. (**c**,**d**) The traces of SD(2) of the deterministic stable fixed point, period cycles, and chaos for the NLARI in (**a**,**b**) without noise for *κ*_**2**_ = 1 and *κ*_**2**_ = 4, respectively. (**e**,**f**) The traces of SD(2) versus the stability coefficient *γ* for the NLARI’ stochastic stable fixed point, period cycles, and chaos when the amplitude indicator *η*_**2**_ is decreased by increasing the restoration coefficient *β* for *κ*_**2**_ = 1 and *κ*_**2**_ = 4, respectively. (**g**,**h**) The traces of SD(2) of the deterministic stable fixed point, period cycles, and chaos for the NLARI in (**e**,**f**) without noise for *κ*_**2**_ = 1 and *κ*_**2**_ = 4, respectively. In (**a**–**d**), the resistance coefficient *α* = 1.1 and *β* ∈ (0.036, 5.76). In (**e**–**h**), *α* ∈ (1, 2) and *β* ∈ (0.001, 0.04).

Next, we revealed the self-similarity and control mechanisms of the NLARI with and without noise in the whole dynamic region with odd and even restoration delays, and clarified the impact of noise on the self-similarity. The sd of the sd similarity-ratio sequence *sd*(*sd*_(*i*,*im*)_) in the aggregation size $i=1,\ldots ,50$ for any given *m*, SD(*m*), was used as a measure of self-similarity level. A near null SD(*m*) represents self-similarity. As an example, Fig. 5 presents a continuum consisting of the traces of SD(2) of the NLARI versus the stability coefficient *γ* in restorative delays of one and four. In noise case, each trace corresponds to the same amplitude indicator. For a given resistance coefficient (Fig. 5a–d), all traces of SD(2) of the stable fixed point tend to zero (long-range dependence) in which a smaller amplitude indicator has a smaller SD(2) and the deterministic stable fixed point has a null SD(2) due to *η*_2_ = 0, whereas all traces of SD(2) of the period cycles and chaos deviate significantly from zero (no self-similarity). For a wide range of the resistance coefficient (Fig. 5e–h), we see the similar traces of SD(2), but the traces of SD(2) for the period cycles and chaos with four restorative delay tend to zero like the stable fixed point (Fig. 5f,h). In summary, the deterministic/stochastic stable fixed point can exhibit self-similarity in which the amplitude indicator controls the level of self-similarity in a negative relationship, while the deterministic/stochastic period cycles and chaos have no self-similarity for an odd restorative delay, but have self-similarity for an even restorative delay.

### Smallest observation scale of fractals

Next, we explored the connection between the observation scale and fractal emergence. A time series in self-regulating systems uncertainly has a relatively large slope indicator and a relatively small amplitude indicator. But examples suggest that enlarging the observation scale may lead to the emergence of self-similarity. For example, it is proposed that, at time scales of at least 103–106 year, sequences and systems tracts are scale-invariant fractal features in the geology of stratigraphic sequences^19^.

**Figure 6:**
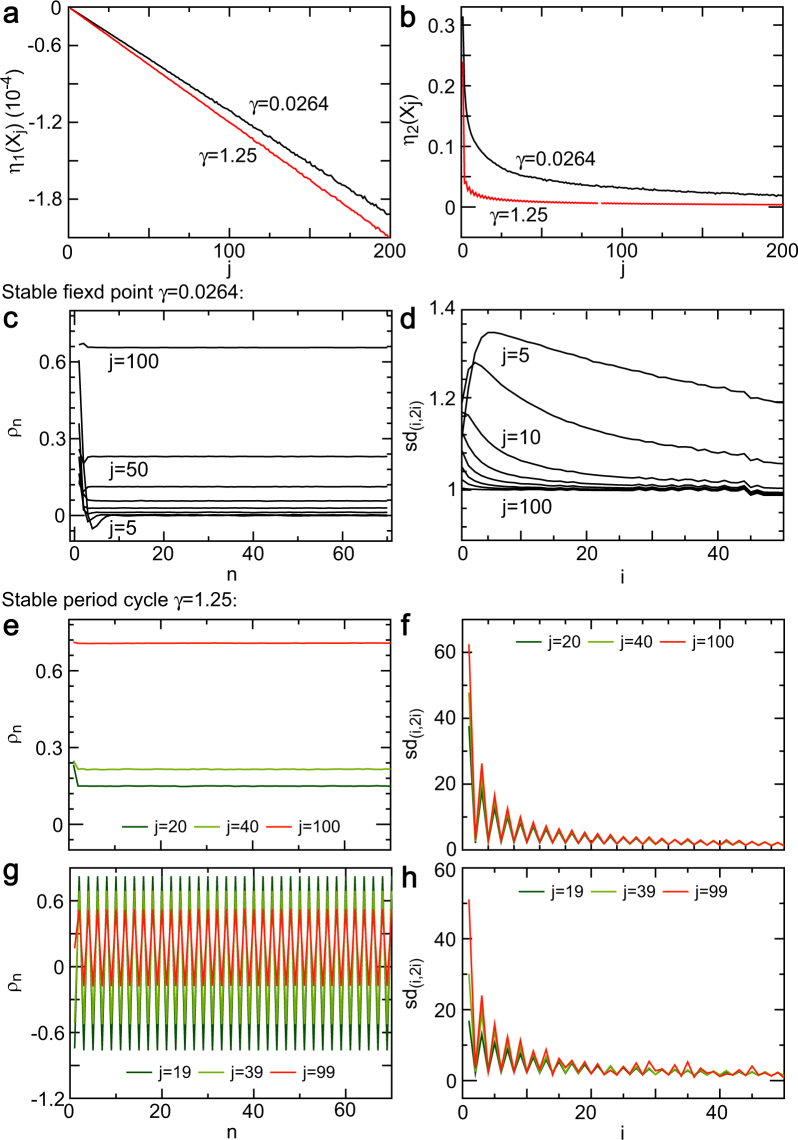
Determining the smallest observation scale of fractals. (**a**) Plot of the absolute slope indicator (|*η*_1_(*X*_*j*_)|) of the aggregation series (*X*_*j*_) draws a declining line as size (*j*) increases in the stable fixed-point region (*γ* = 0.0264) and the stable period-cycle region (*γ* = 1.25). (**b**) Amplitude indicator (*η*_**2**_(*X*_*j*_)) of the aggregation series (*X*_*j*_) rapidly decays as size (*j*) increases in the stable fixed-point region (*γ* = 0.0264) and the stable period-cycle region (*γ* = 1.25). (**c**) Autocorrelation function (ACF) *ρ*_*n*_ of the aggregation series versus the lag *n* changes from rapid decay to almost unchanging in *j* = 5, 10, 15, 23, 30, 40, 50, 100, thus showing long-range dependence. (**d**) Similarity ratio (*sd*_(*i*,**2***i*)_) of the aggregation series plotted versus size (*i*) tends to a horizontal line as size (*j*) increases from *j* = 5 to *j* = 100, thus showing self-similarity. (**e**,**g**) Plot of the sample ACF of the aggregation series versus the lag results in a horizontal line for even number (*j*) and period oscillations odd number (*j*), thus showing long-range dependence, although the dependence is unrelated to the aggregation size (*j*) or increased |*η*_1_(*X*_*j*_)|. (**f**,**h**) Similarity ratio (*sd*_(*i*,**2***i*)_) of the aggregation series versus size (*i*) generates damped oscillations, which are unrelated to size (*j*) or decreased *η*_**2**_(*X*_*j*_).

Enlarging the observation scale of a process means aggregating the time series generated by the process based on equation (7). A long restorative delay of a process can be shortened by aggregating the time series of the process. For this reason, we focused on investigating the case of an one-restorative delay. We calculated the simulation estimates of the slope indicator and amplitude indicator of the aggregated time series at aggregate sizes of 1 to 200 using the data generated by equation (1) in the stable fixed-point and period-cycle regions for a restorative delay of one. As aggregate size increases, the absolute slope indicator increases almost linearly (Fig. 6a), whereas the amplitude indicator decays exponentially (Fig. 6b) in the stable fixed-point region (e.g., *γ* = 0.0264) and the stable period-cycle region (e.g., *γ* = 1.25). The fractal level of the aggregated time series increases as the aggregated size increases in the stable fixed-point region. For instance, the increased absolute slope indicator by aggregation makes the trace of the sample ACF versus size tend toward a horizontal line that shows persistent dependence (Fig. 6c). The similarity ratio plot tends toward a roughly horizontal line which shows self-similarity (Fig. 6d). In the stable period-cycle region, the sample ACF plot of the aggregated time series exhibits roughly horizontal lines for even number sizes (Fig. 6e) and persistent period oscillations for odd number sizes (Fig. 6g). Moreover, the sd similarity ratio plot of the aggregated time series exhibits decay that changes exponentially with damped oscillations for even number sizes (Fig. 6f) and odd number sizes (Fig. 6h). These results indicate that aggregating a time series simultaneously increases the absolute value of the slope indicator and lowers the value of the amplitude indicator, which enhances fractal level of stochastic stable fixed point. However, the aggregations fail to increase self-similarity level of stochastic period cycles, although they can exhibit long-range dependence.

We demonstrated that in the stable fixed-point region there is the smallest observation scale at which long-range dependence and self-similarity can emerge simultaneously. We identified that aggregating a time series could enhance its stability and particularly the stable fixed point structure remained unchanged after aggregation. If a time series is generated by NLARI, then its parameters *θ*_1_, *θ*_2_, and *γ* should lie within the theoretical intervals (−1, 1), (0, 4) and (0, 1) (ref.^49^). We calculated the parameters *θ*_1_, *θ*_2_, and *γ* of equation (1) in the stable fixed-point region for the aggregated time series at size 100. All parameters fell within these theoretical intervals for the stable fixed point at the 97.5% confidence level for single-tailed tests (see Methods).

As shown above, long-range dependence and self-similarity were not equivalent. In the stable fixed-point region, low self-similarity could be accompanied by high long-range dependence or vice versa, when an observation scale was not large enough. For example, gave *ω* = 0.00008, *σ* = 0.05, *α* = 1.1, and *β* = *γ* (4 − 2*α*) for *γ* = 0.02*j* in the period-cycle region for $j=51,\ldots ,153$ and *κ*_2_ = 1, its SACF was close to 70 (the last trace in Fig. 3a), which reflected high dependence, but its SD(2) was far from zero (the second trace in Fig. 5a), which reflected low self-similarity; reversely, in the stable fixed-point region for $j=1,\ldots ,14$ and *κ*_2_ = 4, its SACF was far below 70 (the last trace in Fig. 3b), which reflected low dependence, whereas its SD(2) was close to zero (the second trace in Fig. 5b), which reflected high self-similarity.

### Multi-dimensional fractals

#### Multi-dimensional space-times

Consider a (1 + *k*)-dimensional space-time where a time axis is vertical to the *k*-dimensional space. Let ${\bf{W}}(t)=\{t,{X}_{1}(t),\ldots ,{X}_{k}(t)|t\in R\}$ be a continuous-time point and ${{\bf{W}}}_{t}=(t,{X}_{1t},\ldots ,{X}_{kt}|t=1,2,\ldots )$ be a discrete-time point in the space-time. The integer cases *D* = 1, 2, 3 of fractal dimension correspond to a line segment, a square, and a cube provided by *D* = *log*(*n*^1^)/*log*(*n*) = 1, *D* = *log*(*n*^2^)/*log*(*n*) = 2, and *D* = *log*(*n*^3^)/*log*(*n*), respectively. While the occurrence of a line segment, a square, or a cube implies that extrinsic stochastic forcing lacks or is small so that it can be neglected in the system. In this case, *ω*_1_ = *σ*_1_ = 0 or *ω*_1_/*α*_1_ = *σ*_1_/*β*_1_ = 0, the NLARI process in equation (1) with *κ*_2_ = 1 is a stable fixed point *X*_1*t*_ = *X*_10_ for 0 < *γ*_1_ = *β*_1_(4 − 2*α*_1_) < 1. Thus, *X*_1*t*_ = *X*_10_ can express a line segment for *k* = 1, a square for *k* = 2, and a cube for *k* = 3. That is, the zero values of the slope indicator and the amplitude indictor in a (1 + 1)-, (1 + 2)-, and (1 + 3)-dimensional space-time correspond to the integer cases *D* = 1, 2, 3 of fractal dimensionality, respectively. According to this result, an object with a straight line, a square, or a cube in the real world suggests that the object lies in a stable intrinsic system and the relative strength of extrinsic influence to intrinsic forces is small.

In the (1 + *k*)-dimensional space-time, we introduced the multi-NLARI

9 $${X}_{it}={\omega }_{i}+(2-{\alpha }_{i}){X}_{it-1}-(1-{\alpha }_{i}){X}_{it-2}+{\beta }_{i}\frac{-({X}_{it-{\kappa }_{2}}-{\mu }_{it-{\kappa }_{2}})}{\exp [{({X}_{it-{\kappa }_{2}}-{\mu }_{it-{\kappa }_{2}})}^{2}]}+{\varepsilon }_{it}$$

where *μ*_*it*_ = *X*_*i*0_ + (*ω*_*i*_/*α*_*i*_)*t* if *ε*_*it*_ is white Gaussian noise, ${\varepsilon }_{it}={{\boldsymbol{\epsilon }}}_{it}-{\omega }_{i}$ and ${\omega }_{i}=E({{\boldsymbol{\epsilon }}}_{it})$ for $i=1,\ldots ,k$, and ${{\boldsymbol{\epsilon }}}_{t}=({{\boldsymbol{\epsilon }}}_{1t},\ldots {{\boldsymbol{\epsilon }}}_{kt})$ is an external random disturbance. When the components of the random disturbance are multiplically independent, their mean matrix is given by $diag\,({\omega }_{1},\ldots ,{\omega }_{k})$ and their covariance matrix is given by ${\rm{\Omega }}=diag\,({\sigma }_{1}^{2},\ldots ,{\sigma }_{k}^{2})$. For a simple case of *k* = 3, ***σ*** = 0, and *ω*_*i*_ ≠ 0 for *i* = 1, 2, 3, equation (9) is *X*_*it*_ = *X*_*i*0_ + (*ω*_*i*_/*α*_*i*_)*t* for 0 < *γ*_*i*_ < 1. Then, $\frac{{\alpha }_{1}}{{\omega }_{1}}({X}_{1t}-{X}_{10})=\frac{{\alpha }_{2}}{{\omega }_{2}}({X}_{2t}-{X}_{20})=\frac{{\alpha }_{3}}{{\omega }_{3}}({X}_{3t}-{X}_{30})$—a straight line in the 3-dimensional space; thus, the ${{\bf{W}}}_{t}=(t,{X}_{1t},{X}_{2t},{X}_{3t})$ traces a plane vertical to the 3-dimensional space.

#### Measures and control indicators

According to the above analysis, an ACF near one at large lags implies long-range dependence, while a near null sd of similarity-ratio sequence SD(*m*) for any integer *m* implies self-similarity in 1-dimensional spaces. Thus, we proposed the distance between sample ACF points $({\rho }_{n1},\ldots ,{\rho }_{nk})$ for the lag *n* and a point of one ${(1,\ldots ,1)}_{1\times k}$ at large lags as a measure of dependence, while the distance between sample similarity-ratio points $(SD{(2)}_{i1},\ldots ,SD{(2)}_{ik})$ for the stability coefficient *γ*_*i*_ and the origin ${(0,\ldots ,0)}_{1\times k}$ as a measure of self-similarity in *k*-dimensional spaces. A small distance represents a high level fractal. We introduced the wave indicator ***η*** = (***η***_**1**_, ***η***_**2**_) into (1 + *k*)-dimensional space-times where the slope indicator ${{\boldsymbol{\eta }}}_{1}=({\omega }_{1}/{\alpha }_{1},\ldots ,{\omega }_{k}/{\alpha }_{k})$ and the amplitude indictor ${{\boldsymbol{\eta }}}_{2}=({\sigma }_{1}/{\beta }_{1},\ldots ,{\sigma }_{k}/{\beta }_{k})$. We demonstrated that the wave indicator as the control indicator of 1-dimensional fractals was still valid for multi-dimensional fractals when the components of the external random disturbances were multiplically independent.

**Figure 7:**
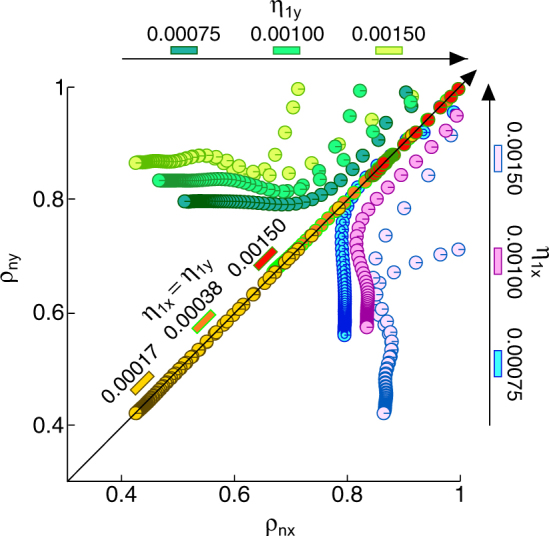
Long-dependence indicator in multi-dimensional spaces. The scatter diagram graphs pairs of sample ACFs $({\rho }_{1x},{\rho }_{1y}),({\rho }_{2x},{\rho }_{2y}),\ldots ,({\rho }_{70x},{\rho }_{70y})$. Their components ${\rho }_{1y},\ldots ,{\rho }_{70y}$ (or ${\rho }_{1x},\ldots ,{\rho }_{70x}$) placed above (or below) the diagonal line at larger lags become larger than 0.8 with rising *η*_1*y*_ (or *η*_1*x*_) from 0.00075 to 0.00150; those lying on the diagonal line at larger lags become larger than 0.8 with rising *η*_1*y*_ or *η*_1*x*_ from 0.00017 to 0.00150; and all the ACF points placed above/below/on the diagonal line at larger lags decay more slowly and the distances between the sample ACF points and the point of one become smaller with rising slope indicator (*η*_1*x*_, *η*_1*y*_). These results indicates that the slope indicator is also a long-dependence indicator in multi-dimensional spaces.

For the slope indicator (*η*_1*x*_, *η*_1*y*_), we investigated the relationship between the slope indicator and the dependence for *k* = 2 in *η*_1*x*_ < *η*_1*y*_ (which corresponds to the area above the diagonal line), *η*_1*x*_ > *η*_1*y*_ (which corresponds to the area below the diagonal line), and *η*_1*x*_ = *η*_*y*_ (which corresponds to the diagonal line). Figure 7 shows the scatter diagram that graphs sample ACF points (*ρ*_*nx*_, *ρ*_*ny*_) for the lag $n=1,\ldots ,70$ at different positive values of the slope indicator (*η*_1*x*_, *η*_1*y*_) in the stable fixed-point range using the data generated by equation (9). We see that the *ρ*_*ny*_ values placed above the diagonal line decay at larger lags more slowly with rising *η*_1*y*_; the *ρ*_*nx*_ values lying below the diagonal line at larger lags decay more slowly with rising *η*_1*x*_; and all the ACF points placed above/below/on the diagonal line at larger lags decay more slowly and the distance between the sample ACF points and the point of one becomes smaller with rising slope indicator (*η*_1*x*_, *η*_1*y*_). This shows that a relatively large absolute slope indicator can lead to long-range dependence in multi-dimensional spaces as seen in 1-dimensional spaces.

**Figure 8:**
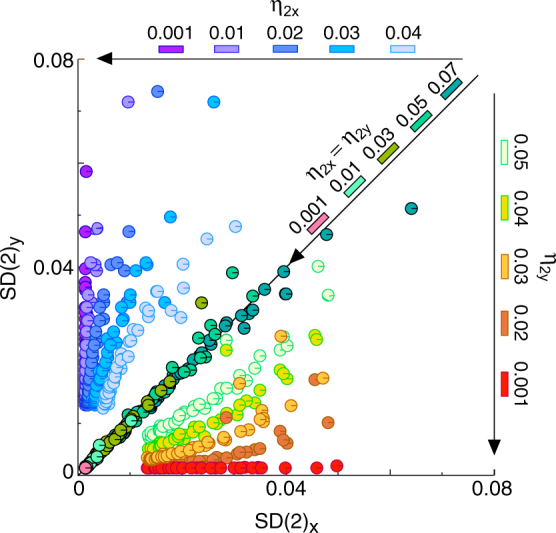
Self-similarity indicator in multi-dimensional spaces. The scatter diagram graphs pairs of the sd series of sample similarity-ratio sequence *SD*(2) = (*SD*(2)_*x*_, *SD*(2)_*y*_) across the stable fixed-point range at different values of the amplitude indicator (*η*_2*x*_, *η*_2*y*_). The *SD*(2)_*x*_ values placed above the diagonal line become smaller as *η*_2*x*_ approaches 0; the *SD*(2)_*y*_ values lying on the diagonal line become smaller as *η*_2*y*_ approaches 0; and all the *SD*(2) points placed above/below/on the diagonal line become smaller and the distances between the *SD*(2) points and the origin are close to 0 as (*η*_2*x*_, *η*_2*y*_) approaches 0. These results show that the amplitude indicator acts as a self-similarity indicator in multi-dimensional spaces.

For the amplitude indicator (*η*_2*x*_, *η*_2*y*_), we focused on the relationship between the amplitude indicator and the self-similarility for *k* = 2 in the area above/below/on the diagonal line. Figure 8 shows the scatter plot of the sd series of the sample similarity-ratio sequence *SD*(2) = (*SD*(2)_*x*_, *SD*(2)_*y*_) across the stable fixed-point range at different values of the amplitude indicator. It is seen that the *SD*(2)_*x*_ values placed above the diagonal line are close to zero as the *η*_2*x*_ decreases to zero; the *SD*(2)_*y*_ values lying below the diagonal line are close to zero as the *η*_2*y*_ decreases to zero; and all the *SD*(2) points placed above/below/on the diagonal line are close to the origin, so that the distances between the *SD*(2) points and the origin become smaller as the amplitude indicator (*η*_2*x*_, *η*_2*y*_) decreases to zero. These results show that the amplitude indicator can still act as a control indicator of self-similarity in multi-dimensional spaces.

### Empirical evidence for control indicators

**Figure 9:**
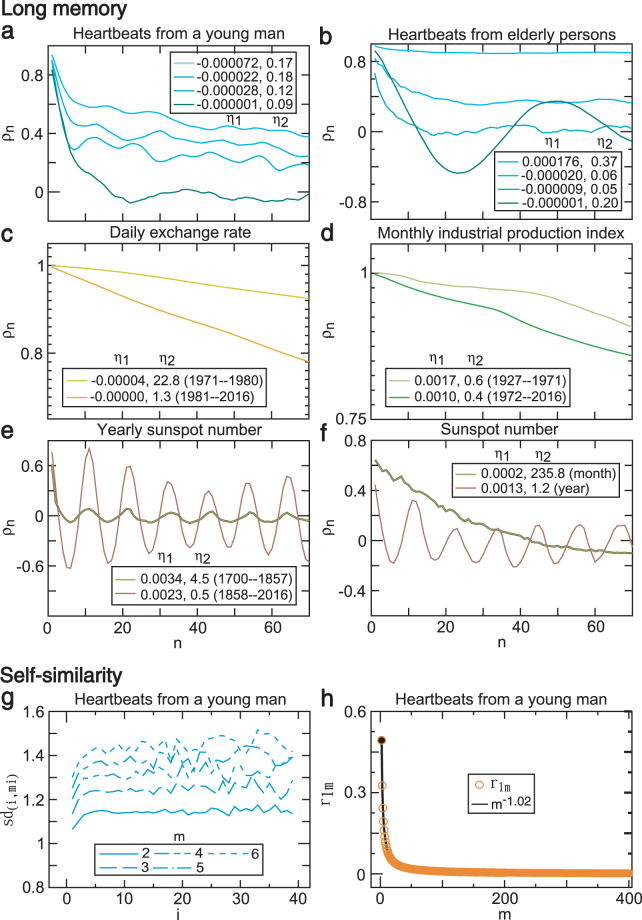
Real world evidence of fractals and control indicators. (**a**) Sample autocorrelation function (ACF) *ρ*_*n*_ of four heartbeat series from a young man slowly decay as the lag (*n*) increases (the dependence rises) as the absolute slope indicator |*η*_1_| and the amplitude indicator *η*_2_ increase. (**b**) Decay of the sample ACF of the heartbeats from elderly persons (the top three lines) reduces (the dependence increases) as the values of |*η*_1_| and *η*_2_ increase, while the fluctuating decay of the sample ACF of the heartbeats (bottom) with the smallest |*η*_1_| and the second largest *η*_2_ in this group is slow. (**c**) Trace of the sample ACF of the U.S./U.K. foreign exchange rate for the period 1971–1980 decays more slowly (the higher dependence level) than that for the period 1981–2016; the former has a larger |*η*_1_| and *η*_2_ than the latter. (**d**) Decay of the sample ACF of the U.S. industrial production index for the period 1927–1971 decays more slowly (the higher dependence level) than that for the period 1972–2016, the former has larger |*η*_1_| than that of the latter and both have the same *η*_2_. (**e**) Fluctuating decay of the sample ACF, the yearly mean total sunspot number for the period 1858–2016 is much slower (the higher dependence level) than that for the period 1700–1857; the *η*_2_ for the latter is much larger than that of the former and the *η*_1_ is slightly greater than zero. (**f**) For the period 1749–2016, the trace of the sample ACF, which is the yearly mean total sunspot number, exhibits persistent oscillations, whereas the trace of the sample ACF, which is the monthly mean total sunspot number, rapidly declines, and the *η*_2_ in the yearly data is much larger than that in the monthly data and the *η*_1_ is slightly greater than zero. (**g**) Plotting the standard deviation similarity ratios *sd*_(*i*,*im*)_ versus size (*i*) results in a roughly horizontal line when *m* = 2 and fluctuations occur along the horizontal lines when *m* = 3, 4, 5, 6 because the sample length is not long enough to exhibit the self-similarity of the system. (**h**) Average of the lag one similarity ratios (*r*_1*m*_) of *r*_(*i*,*im*)_ over size (*i*) follows a power function with power −1.02.

Here we provided empirical evidence that (i) a relatively large absolute slope indicator could lead to long-range dependence; (ii) the amplitude indicator could also influence the dependence level and pattern; and (iii) a relatively small amplitude indicator could lead to self-similarity. The data sets include heartbeats, exchange rates, industrial production index, and sunspot numbers, which are regarded as the outputs of self-regulating systems. To avoid potential sample size effects on the dependence, all the sample ACF plots in each panel were obtained based on the same sample size. We compared the relationship of the dependence level (decay rate of ACF) with the control parameters *η*_1_ and *η*_2_ in the same object/variable and different time periods (Fig. 9a), the same variable and different objects (Fig. 9b), the same variable and different time periods (Fig. 9c–e), and the same variable/time period and different frequencies (Fig. 9f). A slower decay ACF plot corresponded to a larger absolute slope indicator in all cases and a larger amplitude indicator in most cases but an over large amplitude indicator (Fig. 9f). These empirical results support our proposition. We further calculated the sample sd similarity ratios, the lag *h* autocovariance similarity ratios, and their average similarity ratio using clinical heartbeat observations (52200 data points). The similarity ratio plot (*sd*_(*i*,*im*)_) at an aggregate size of two displayed a roughly horizontal line, although the similarity ratio fluctuated somewhat at other aggregate sizes (Fig. 9g) and the average similarity ratio (*r*_1*m*_) generated a power function with power −1.02 (Fig. 9h), suggesting a weak self-similarity. This result occurred because the amplitude indicator of the original heartbeat series was a relatively large value (*η*_2_ = 0.3136) and the sample size was not large enough to lower the amplitude indicator by aggregating the series. Our results support previous findings of heartbeat fractals^25,33,53–57^.

## Discussion

Compared with widely held expectations, we discovered that fractal signals in self-regulating systems could be measured by an integer-dimensional space-time. Along the evolutionary route from a stable fixed point to chaos of NLARI in the 1 + 1-dimensional space-time, we demonstrated that (i) a stochastic stable fixed point could exhibit long-range dependence and self-similarity, while a deterministic stable fixed point could exhibit self-similarity but usually no long-range dependence; (ii) the wave indicators primarily controlled the level of dependence (self-similarity) for a stable fixed point: a larger absolute slope (smaller amplitude) indicator led to a higher level of positive dependence (self-similarity), and a relatively large amplitude indicator or an even restorative delay could make the dependence oscillate; (iii) sufficiently aggregating a time series, which was equivalent to enlarging observation scale, in the stable fixed-point region provided both a small amplitude indicator and a large absolute slope indicator, which led to self-similarity and long-range dependence; (iv) the slope (amplitude) indicator was determined by the ratio of the disturbance mean (standard deviation) to the resistance (restoration) coefficient, which reflected the relative strength of extrinsic versus intrinsic forces to the system, and was positively correlated with the sample sd of the data; and (v) period cycles and chaos could exhibit long-range dependence and usually no self-similarity for an odd restorative delay, but could have both long-range dependence and self-similarity for an even restorative delay; and their level of long-range dependence (self-similarity) relied on the intrinsic resistance, restoration, and regulatory delay and the effects of extrinsic stochastic forcing could be neglected.

In this article, we extended the wave indicators into multi-dimensional space-times by introducing multi-dimensional NLARI process. We demonstrated that the wave indicators, which reflected the relative strength of extrinsic versus intrinsic forces to the system, could still valid for the multi-dimensional fractals when the components of the external random disturbances were multiplically independent. But the multi-dimensional fractals and control mechanisms in a general case for multiple correlation among the external disturbances remain an important direction of future work.

The present findings will deepen understanding of the physical mechanisms underlying the sd, scales, and fractals. For example, the finding (iv) implies that a low sd reflects a small extrinsic disturbance if there are not greatly intrinsic forces. This inference helps us explain the reason of the island rule^58^. The main difference between an island and a mainland lies in that the island sustains less extrinsic disturbance than the mainland; thus, the island has a small |*η*_1_| or/and *η*_2_ and then a low sd. The low sd provides an explanation for the ecological pattern of large species tending toward smaller body size and small species tending toward larger body size in island populations. Again, when the intrinsic regulation is in the stable fixed-point region and unchanged, the extrinsic effect is responsible for dependence. Extreme events result in a greatly increased disturbance mean or sd, followed by a greatly increased absolute slope indicator, thereby leading to long-range dependence, which explains why the emergence of extreme events is closely related to long-range dependence^59,60^. On the other hand, when there is no great extrinsic disturbance, we have the following results: i) a higher dependence reflects a smaller resistive or restorative force (e.g., long-range dependence of the U.S./U.K. foreign exchange rate from 1971–1980 was due primarily to a small restorative force by *β* = 0.000085, which resulted in a large *η*_2_ = 22.8). ii) A higher self-similarity suggests a larger restorative force, whereas the highest self-similarity level is represented by a straight line and suggests no or weak extrinsic disturbances. iii) A large value of the smallest observation scale suggests a weak restorative force (e.g., the smallest observation scale in stratigraphic sequences is thought to be 10^3^–10^6^ yr.^19^, suggesting a very weak restorative force). iv) South Africa’s coast with a near straight line (*D* = 1.02 has been observed, see ref.^61^) may be due to a small |*η*_1_| because the Great Escarpment parallel to the coast of Southern Africa may have a large resistance coefficient caused by hard and resistant rock. We expected that the fractal control mechanisms and physical implications can provide a novel approach for product development with desirable fractal levels by regulating the object’s parameters and aggregation size.

A comparison of the wave indictors with the scaling exponents helps us to effectively evaluate and measure fractal signals. The typical scaling exponent, the Hurst exponent, measures self-similarity and long-rang dependence in which short-range dependence for 0 < *H* < 1/2, intermediate memory or long-range negative dependence for −1/2 < *H* < 0, and long-range dependence for 1/2 < *H* < 1. The most commonly used estimating the Hurst exponent includes R/S analysis and DFA. R/S analysis has been introduced by Hurst^62^, redefined by Mandelbrot^63^, and modified by Lo^51,64^. DFA has been invented by Peng *et al*.^9,65^ and it has successfully been applied to diverse fields such as DNA^66–68^. In contrast with the Hurst exponent as a single fractal scale, the two wave indictors jointly control fractal level: the amplitude indicator controls self-sterility, the slope indicator controls positive dependence, and the amplitude indicator or/and an even restorative delay make negative dependence and the dependence oscillate. In this study, we showed a positive relation between the slope indicator and the Hurst exponent in the stable fixed-point region using DFA and R/S analysis. The positive relation may help us to compensate for this limitation of this method due to the lack of a precise formulation of ACF. The autoregressive fractionally integrated moving average process also has no precise formulation of ACF for lag orders higher than two^69^. Measurements of fractal dimensions are affected by various methodological issues, which increases the difficulty of comparing results obtained using different methods^70–73^. How the positive relation is used to exactly identify long-range positive dependence remains in future work.

Another limitation of this method is invalid for a rapidly changing system, because the linearization of the resistance function requires a small *E*|*X*_*t*_ − *X*_*t*−1_| (ref.^47^). It needs to be pointed out that the stability of the NLARI’ deterministic system is not globally stable^48^ so that certain assumptions are required to ensure an asymptotically unbiased and consistent parameter estimator^49^. Processing to reduce the magnitude of the data, for example, calculating the logarithm often meets the above required conditions because it brings about a small sd and a small *E*|*X*_*t*_ − *X*_*t*−1_| by *Y*_*t*_ − *Y*_*t*−1_ = log(*X*_*t*_/*X*_*t*−1_). When the data processing cannot reduce the change rate of data, the self-regulating system can only be specified by

10 $${X}_{t}=\omega +2{X}_{t-1}-{X}_{t-2}-\alpha \frac{{X}_{t-{\kappa }_{1}}-{X}_{t-{\kappa }_{1}-1}}{{e}^{{({X}_{t-{\kappa }_{1}}-{X}_{t-{\kappa }_{1}-1})}^{2}}}-\beta \frac{{X}_{t-{\kappa }_{2}}-{\bar{X}}_{t-{\kappa }_{2}}}{{e}^{{({X}_{t-{\kappa }_{2}}-{\bar{X}}_{t-{\kappa }_{2}})}^{2}}}+{\varepsilon }_{t}$$

where ${\bar{X}}_{t}=\frac{1}{t}\,{\sum }_{j=1}^{t}\,{X}_{j}$ (see Supplementary information). Although we used the Krylov-Bogoliubov averaging method^74^ to obtain the approximate solutions of equation (10), this problem remains open.

## Methods

### Statistical analysis and calculations

#### Estimation

Let Δ*Y*_*t*_ = *Y*_*t*_ − *Y*_*t*−1_. Equation (2) can be written as follows:

11 $${\rm{\Delta }}{Y}_{t}={\theta }_{1}{\rm{\Delta }}{Y}_{t-1}+{\theta }_{2}\frac{-{Y}_{t-{\kappa }_{2}}}{{e}^{{Y}_{t-{\kappa }_{2}}^{2}}}+{\varepsilon }_{t}$$

for $t=1,2,\ldots ,T$. The parameters for equations (1) and (2) were estimated via a simulation and an empirical study using the following procedure. The ordinary least squares (OLS) estimates ${\hat{\theta }}_{1}$, ${\hat{\theta }}_{2}$, and $\hat{\sigma }$ for equation (11) were obtained with data generated using ${Y}_{t}={X}_{t}-\hat{a}-\hat{b}t$ where ${\mu }_{t}\approx \hat{a}+\hat{b}t$, $\hat{a}$ and $\hat{b}$ were determined by the OLS regression line ${\hat{X}}_{t}=\hat{a}+\hat{b}t$ with data *X*_*t*_ generated using equation (1) for simulations and *X*_*t*_ = log(real data) for estimations. Then, $\hat{\alpha }=1-{\hat{\theta }}_{1}$, $\hat{\beta }={\hat{\theta }}_{2}$, ${\hat{\eta }}_{1}=\hat{b}$, and ${\hat{\eta }}_{2}=\hat{\sigma }/{\hat{\theta }}_{2}$. The simulation parameter values were provided or estimated using simulated data generated by equation (1) with ${\hat{\theta }}_{0}=\hat{b}(1-{\hat{\theta }}_{1})$, ${\hat{\theta }}_{1}$, ${\hat{\theta }}_{2}$, $\hat{\sigma }$ and ${\hat{\mu }}_{t-1}=\hat{a}+\hat{b}(t-1)$. Each simulation parameter was usually an average of 3000 realizations; however, the simulation ACF in Fig. 2d and were calculated once in each iteration, and their fluctuating traces are displayed.

#### Hypothesis testing

Testing NLARI: The standard *t* and *F* statistics based on ${\hat{\theta }}_{1}$, ${\hat{\theta }}_{2}$, and $\hat{\gamma }$ have the standard normal limiting distributions for *γ* > 0 under the given assumptions^49^. In this article, we showed that the standard *t*-statistic ${t}_{{\hat{\eta }}_{2}}$ has a standard normal limiting distribution, which ensures the effectiveness of ${\hat{\eta }}_{2}$. We defined the statistic ${t}_{{\hat{\eta }}_{{\bf{2}}}}=({\hat{\eta }}_{{\bf{2}}}-{\eta }_{{\bf{2}}})/({\hat{\eta }}_{{\bf{2}}}^{{\bf{2}}}\sqrt{{s}^{{\bf{22}}}})$ where

12 $${t}_{{\hat{\eta }}_{2}}=(\frac{{\hat{\theta }}_{2}-{\theta }_{2}}{\hat{\sigma }\sqrt{{s}^{22}}}-\frac{{\theta }_{2}}{\sigma \hat{\sigma }\sqrt{{s}^{22}}}(\hat{\sigma }-\sigma ))\times (-\frac{{\eta }_{2}}{{\hat{\eta }}_{2}})$$

where $s$^22^ is the row 2 and column 2 element of ${Y}_{1,t-1}=({\rm{\Delta }}{Y}_{t},-\,{Y}_{t-1}{e}^{-{Y}_{t-1}^{{\bf{2}}}})$, ${(\frac{1}{T}Y^{\prime} Y)}^{-1}$, and $Y=({Y}_{1,0}^{^{\prime} },\ldots ,{Y}_{1,T-1}^{^{\prime} })^{\prime}$. According to Proposition 3.4 in ref.^49^, $({\hat{\theta }}_{2}-{\theta }_{2})\,{(\hat{\sigma }\sqrt{{s}^{{\bf{22}}}})}^{-1}\mathop{\to }\limits^{d}N(0,1)$ as $T\to \infty$ under certain assumptions. Combining equation (11) derives ${t}_{{\hat{\eta }}_{{\bf{2}}}}\mathop{\to }\limits^{d}N(0,1)$ as $T\to \infty$. These assumptions are required because the stability of the fixed point of the NLARI process is not globally asymptotically stable^48^. These assumptions are usually met for a relatively small *σ* according to the simulation study^49^. To achieve such a relatively small *σ*, the data are processed to reduce the magnitude, which often helps meet these assumptions (see Supplementary Information). A sample size smaller than 1800 may underestimate the level of dependence so each case had the sample size *T* ≥ 1800 in Figs 2, 3, 4, 5, 6, 7 and 8.

Testing aggregated NLARI: We identified that the structure of the stable fixed point was unchanged after aggregating a time series. Let *η*_1_(*X*_*j*_) and *η*_2_(*X*_*j*_) be the simulated estimations *η*_1_ and *η*_2_ of the aggregated series *X*_*j*_ at *j* = 1 to 200 with the data generated by equation (1) in the stable fixed-point region 0 < *γ* < 1 and period-cycle region $1 < \gamma  < \sqrt{e}$ for *κ*_1_ = *κ*_2_ = 1. The original data were generated by equation (1) using the same parameter values of Fig. 6a: *θ*_0_ = −4.4753 × 10^−7^, *θ*_1_ = 0.50275, *θ*_2_ = 0.07940, *γ* = 0.0264, and *σ* = 0.02493. The estimated parameters for the aggregated series *X*_*j*_ for *j* = 100 were ${\hat{\theta }}_{1}=0.0072$, ${\hat{\theta }}_{2}=0.9867$, and $\hat{\gamma }=0.4898$, which corresponds to the confidence intervals (−0.0812, 0.0914), (0.8628, 1.1048), and (0.4291, 0.5497). These values fell within the theoretical intervals (−1, 1), (0, 4) and (0, 1) for the stable fixed point at the 97.5% confidence level for single-tailed tests.

#### Multi-dimensional fractal calculations

For Fig. 7, we calculated the ACF points (*ρ*_*nx*_, *ρ*_*ny*_) for the lag $n=1,2,\ldots ,70$ at different values of the slope indicator by *η*_1*nx*_ = *σ*_*x*_/*α*_*nx*_ and *η*_1*ny*_ = *σ*_*y*_/*α*_*ny*_ using the data generated by equation (9) where *ω*_*x*_ = *ω*_*y*_, *σ*_*x*_ = *σ*_*y*_, and *β*_*x*_ = *β*_*y*_. For Fig. 8, we calculated the values of *SD*(2) = (*SD*(2)_*x*_, *SD*(2)_*y*_) at the stability coefficient *γ*_*i*_ = 0.02*i* for $i=1,\ldots ,49$ and the amplitude indicator (*η*_2*ijx*_, *η*_2*ijy*_) for $j=1,\ldots ,5$ where *η*_2*ijx*_ = *σ*_*jx*_/*β*_*ix*_, *η*_2*ijy*_ = *σ*_*jy*_/*β*_*iy*_, *α*_*x*_ = *α*_*y*_, and *β*_*ix*_ = *β*_*iy*_ = (4 − 2*α*_*x*_)*γ*_*i*_, which were denoted by *SD*(2)_*ij*_ = (*SD*(2)_*ijx*_, *SD*(2)_*ijy*_). The values of *SD*(2) across the stable fixed-point range (0, 1) were realized by letting *γ*_*i*_ = 0.02*i* for $i=1,\ldots ,49$ which correspond to the stability coefficients ranging from 0.02 to 0.98, while the *SD*(2) at different values of the amplitude indicator were realized by letting *σ*_*jx*_ get different values for a given *σ*_*jy*_ and by letting *σ*_*jy*_ get different values for a given *σ*_*jx*_ (for details of all parameters see Supplementary information).

### Algorithms

The algorithms used in this article cover five areas: Initialization, Estimation, Aggregation, Generation, and Computation as follows:

#### Initialization

Provide the number of repeats *N* (3000 times in this paper), aggregation size *m*, sample size *T*, initial values *X*_0_ and *X*_1_, and the parameter values *α*, *β*, *ω*, *σ*.

#### Generation

Produce the simulated data *X*_*t*_ using equation (1).

#### Aggregation

Calculate the aggregation series at size *m*: $${X}_{m}=({X}_{t}^{(m)}:t=1,\ldots ,[T/m])$$ where $${X}_{t}^{(m)}=\frac{1}{m}$$$${\sum }_{k=1}^{m}\,{X}_{(t-1)m+k}$$.

#### Estimation

Calculate the regression line *X*_*t*_ = *a* + *bt* + *u*_*t*_ using the data *X*_*t*_ (real data or the simulated data) to determine the OLS estimates $\hat{a}$ and $\hat{b}$. Then, estimate equation (10) to determine the OLS estimates ${\hat{\theta }}_{1},\,{\hat{\theta }}_{2}$, and $\hat{\sigma }$ using ${Y}_{t}={X}_{t}-\hat{a}-\hat{b}t$ and Δ*Y*_*t*_ = *Y*_*t*_ − *Y*_*t*−1_ and get $\hat{\alpha }=1-{\hat{\theta }}_{1}$, $\hat{\beta }={\hat{\theta }}_{2}$, $\hat{\omega }={\hat{\theta }}_{0}=\hat{b}(1-{\hat{\theta }}_{1})$, ${\hat{\eta }}_{1}=\hat{b}$, ${\hat{\eta }}_{2}=\hat{\sigma }/{\hat{\theta }}_{2}$, and ${\hat{\mu }}_{t-1}=\hat{a}+\hat{b}(t-1)$.

#### Computation

For long-range dependence, calculate the sample ACF *ρ*_*n*_ = *r*_*n*_/*r*_0_, $${r}_{n}={r}_{n}(X)={\sum }_{t=1}^{T-n}\,({X}_{t}-\bar{X})$$$$({X}_{t+n}-\bar{X})$$, $$\bar{X}=\frac{1}{T}\,{\sum }_{t=1}^{T}\,{X}_{t}$$, and sample $${\rm{SACF}}({\rm{N}})={\sum }_{n=1}^{N}\,|{\rho }_{n}|$$ for *N* = 70. For self-similarity, calculate the sample autocovariance *r*_*h*_(*X*_*j*_) where $${X}_{j}=({X}_{t}^{(j)}:t=1,\ldots ,[T/j])$$ and $${X}_{t}^{(j)}=\frac{1}{j}\,{\sum }_{k=1}^{j}\,{X}_{(t-1)j+k}$$; the similarity ratios *r*_*h*(*i*,*im*)_ and *sd*_(*i*,*im*)_; the standard derivation of the sd similarity ratio sequence SD(2); and the average of the similarity ratios $${r}_{hm}=\frac{1}{n}\,{\sum }_{i=1}^{n}\,{r}_{h(i,im)}$$ over the lag $$h=0,1,\ldots ,30$$, the aggregation sizes $$i=1,\ldots ,50$$ and the similarity-ratio size $$m=2,\ldots ,20$$ for Fig. 4 and $$h=0,\,i=1,\ldots ,50$$, and *m* = 2 for Fig. 5.

Figure 1. 1st Step: Initialization $\Rightarrow$ 2nd Step: Generation. Figures 2, 3, 6, 7 and 8. 1st Step: Initialization $\Rightarrow$ 2nd Step: Generation $\Rightarrow$ 3rd Step: Computation: Repeating the 2nd Step and 3rd Step produces the average, which is the simulation value. Figures 4, 5, 6, 7 and 8. 1st Step: Initialization $\Rightarrow$ 2nd Step: Generation $\Rightarrow$ 3rd Step: Aggregation $\Rightarrow$ 4th Step: Estimation $\Rightarrow$ 5th Step: Computation: Repeating the 2nd Step, 3rd Step, 4th Step, and 5th Step produces the average, which is the simulation value. Figure 9a–f. 1st Step: Estimation $\Rightarrow$ 2nd Step: Computation. Figure 9g,h. 1st Step: Aggregation $\Rightarrow$ 2nd Step: Estimation $\Rightarrow$ 3rd Step: Computation.

### Actual data

The data used in Figs 1, 2, 3, 4, 5, 6, 7 and 8 were generated by computer simulations based on the NLARI model in equation (1), whereas the data used in Fig. 9 were real data. Figure 9a: Heartbeats (record 162659, man, 32 years old, no significant arrhythmias) autocorrelation traces from top to bottom: 37301–39100, 8501–10300, 18001–19800, 1301–3100 records. Figure 9b: Heartbeats from healthy elderly persons, from top to bottom: 3601–5400 (f1o09), 1–1800 (f1o08), 1801–3600 (f1o09), and 1801–3600 (f1o04). Figure 9c: Daily U.S./U.K. foreign exchange rate (DEXUSUK) in 1971:01:30–1980:12:31 and 1981:01:31–2016:12:29. Figure 9d: Monthly U.S. industrial production index (INDPRO) in 1927:1–1971:12 and 1972:1–2016:12. Figure 9e: Yearly sunspot number in 1700–1857 and 1858–2016. Figure 9f: Yearly/monthly sunspot number in 1749–2016. Figure 9g,h: 1299–53298 (record 16265, 52200 points).

Data are available at the MIT-BIH Normal Sinus Rhythm Database^75^ (Fig. 9a,b,g,h); Federal Reserve Economic Data database, https://fred.stlouisfed.Org/series (Fig. 9c,d). The monthly mean total sunspot number [1/1749 - now] and yearly mean total sunspot number [1700 - now], http://www.sidc.be/silso/datafiles (Fig. 9e,f).

## Electronic supplementary material

Supplementary Information
