# Supplementary material for: Integer-dimensional fractals of nonlinear dynamics, control mechanisms, and physical implications
Source: Sci Rep. 2018 Jul 9;8:10324. doi: 10.1038/s41598-018-28669-3 (PMC6037749; doi:10.1038/s41598-018-28669-3)
Supplement: Supplementary file 1 — Supplementary Information [file 41598_2018_28669_MOESM1_ESM.pdf]

Supplementary calculations and modeling for "Integer-dimensional fractals of nonlinear dynamics, control mechanisms, and physical implications"

By Zonglu He
Faculty of Social Information Science
Hiroshima Bunka Gakuen University
3-3-20 Heiseigahama Saka, Akigun, Hiroshima 731-4312, Japan

May 15, 2018

Abstract

1 Derivation and physical meaning of NLARI

We say that a system is a stochastic resilient system if it responds flexibly to external disturbances and tends to return to equilibrium by resisting damage and recovering quickly. Consider the system that experiences a resistive force (f) to hinder a fast change and a restorative force (g) to diminish the deviation from the equilibrium caused by an exogenous stochastic or uncertain disturbance (ϵ). Newton's second law says that a net force results in a change of momentum: $F = f + g + \epsilon = d(m\dot{x})/dt$ of which $F = m\ddot{x}$ is a special case only if mass m is constant, where $\dot{x}$ is the velocity (or change) and $\ddot{x}$ is the acceleration at position x (or the rate of the change in variable x). Without a loss of generality, let $m = 1$; thus, $f + g + \epsilon = \ddot{x}$.

The resistance force is a function of velocity. It can be showed that the resistance force should satisfy the conditions $\dot{x}f(\dot{x}) < 0$ for $\dot{x} \neq 0$ and $f(\dot{x}) = -f(-\dot{x})$ to reflect the nature of a resistance force. Assume that the function f is infinitely differentiable at the origin. If the velocity is relatively slow, we can write $f(\dot{x}) \approx f(0) + f'(0)\dot{x}$ according to Taylor expansion. Because $f(\dot{x}) = -f(-\dot{x})$, thus $f(0) = 0$. Then, we obtain the approximation $f(\dot{x}) = f'(0)\dot{x}$. Because $\dot{x}f(\dot{x}) = f'(0)\dot{x}^2$, thus $f'(0) < 0$ provided by $\dot{x}f(\dot{x}) < 0$ for $\dot{x} \neq 0$. Let $\alpha = -f'(0)$. Hence $\alpha > 0$. It can be proved that the relative restorative coefficient or the stability coefficient γ is given by $\gamma = \beta/(4 - 2\alpha)$ (Far East J. Dynamical Syst. 21, 1–32, 2013). Any negative stability coefficient lacks physical meaning. For this reason, we only consider the case of $\alpha < 2$. We call α the resistance coefficient where $0 < \alpha < 2$.

The restoring force as a variable force that gives rise to an equilibrium in a physical system is a function of the deviation from the mean (expectation). Thus, it is expressed by $g(x - \mu)$. Assume that g is absolutely integrable on $(-\infty, \infty)$ to avoid an explosive solution, and meets the condition $xg(x) < 0$ for $x \neq 0$ to reflect the nature that a restoring force brings the system back down- or up-toward equilibrium after it has been up or down perturbed away from the equilibrium. Let the function $g(x) = -\beta x \exp\left(-x^2\right)$ for $\beta > 0$, which satisfies the conditions required, τ_1 and τ_2 denote delays in the resistance and the restoration, respectively. Then, a stochastic resilient system can be specified by the following continuous NLARI process

$$\ddot{x}(t) = -\alpha\dot{x}(t - \tau_1) + \beta\frac{-\left(x(t-\tau_2) - \mu(t-\tau_2)\right)}{\exp\left(\left(x(t-\tau_2) - \mu(t-\tau_2)\right)^2\right)} + \epsilon(t) \tag{S1}$$

To treat time series data, equation (S1) is discredited by the following. Let ι be the interval of the time series, κ_1 and κ_2 be the integers of τ_1 and τ_2 with $\tau_{1,2} \geqslant \iota$. Denote $X_t = x(t\iota)$ and $\epsilon_t = \epsilon(t\iota)$. For $\iota = 1$, $\ddot{x}(t) \approx X_t - 2X_{t-1} + X_{t-2}$, $\dot{x}(t - \tau_1) \approx X_{t-\kappa_1} - X_{t-\kappa_1-1}$, $x(t - \tau_2) \approx X_{t-\kappa_2}$, and $\mu(t - \tau_2) \approx \mu_{t-\kappa_2} = E\left(X_{t-\kappa_2} \mid X_0, X_{-1}, \cdots, X_{1-\kappa_2}\right)$. Let $\omega = E(\epsilon_t)$ and $\varepsilon_t = \epsilon_t - \omega$. Substituting

them into equation (S1) yields the NLARI process

$$X_t = \theta_0 + (1+\theta_1) X_{t-1} - \theta_1 X_{t-2} + \theta_2 \frac{-(X_{t-\kappa_2} - \mu_{t-\kappa_2})}{\exp\left((X_{t-\kappa_2} - \mu_{t-\kappa_2})^2\right)} + v_t$$

$$\text{with } \theta_0 = \begin{cases} \frac{\omega}{1+\alpha} \\ \omega \end{cases}, \theta_1 = \begin{cases} \frac{1}{1+\alpha} \\ 1-\alpha \end{cases}, \theta_2 = \begin{cases} \frac{\beta}{1+\alpha} \\ \beta \end{cases}, v_t = \begin{cases} \frac{\varepsilon_t}{1+\alpha} & \text{if } \kappa_1 = 0 \\ \varepsilon_t & \text{if } \kappa_1 = 1 \end{cases} \tag{S2}$$

where $\alpha > 0$ is the resistance coefficient, $\beta > 0$ is the restoration coefficient, κ_1 and κ_2 are time delays in the resistance and the restoration, respectively, and ω and σ^2 are the mean and variance of exogenous disturbance ϵ_t, respectively; and a class of the NLARI processes can be obtained by specifying $g(x)$ using various restoration functions. Let $Y_t = X_t - \mu_t$. When the random variable ε_t is white Gaussian noise, we have $Y_t = X_t - X_0 - (\omega/\alpha)\, t$. Equation (S2) can be written as

$$Y_t = (1+\theta_1) Y_{t-1} - \theta_1 Y_{t-2} + \theta_2 \frac{-Y_{t-1}}{\exp\left({Y_{t-1}}^2\right)} + \varepsilon_t \tag{S3}$$

(for details see Far East J. Theor. Stat. 23, 31–50, 2007; and Far East J. Dynamical Syst. 21, 1–32, 2013).

2 Calculations and parameters

In Fig. 1a, $\kappa_2 = 1$; $\omega = 0$; $\sigma = 0$; $\alpha = 1.1$; $\beta = (4-2\alpha)\gamma$; $\gamma = 0.1, 0.9, 1.2, 1.6, 5$. In Fig. 1b, $\kappa_2 = 4$; $\omega = 0$; $\sigma = 0.05$; $\alpha = 1.1$; $\beta = (4-2\alpha)\gamma$; and $\gamma = 0.1, 0.2, 0.5, 1.1, 1.65$. In Fig. 1, $\kappa_1 = 1$ and $T = 200$.

In Fig. 2a, $\omega = 0$; $\alpha = 1.14$; $T = 1800$; and 50000 realizations. In Fig. 2b, $\omega = 0.00600, 0.00065, 0.00042, 0.00030, 0.00022$; $\sigma = 0.05$; $\alpha = 1.40, 1.35, 1.30,$ 1.25, 1.20; and $\beta = 0.1$. In Fig. 2c, $\omega = 0$; $\sigma = 0.20, 0.08, 0.07, 0.06, 0.05$; $\alpha = 0.2$; and $\beta = 0.14, 0.10, 0.09, 0.08, 0.07$. In Fig. 2d, $\omega = 0.00019$; $\sigma = 0.005, 0.01, 0.02, 0.03$; $\alpha = 0.095$; and $\beta = 0.1$. In Fig. 2e, $\omega = 0.00019$; $\sigma = 0.03$; $\alpha = 0.5, 0.42, 0.25, 0.1$; $\gamma = 0.7$; and $\beta = \gamma(4-2\alpha)$. In Fig. 2f, $\omega = 0.0005$, $\sigma = 0.05$, $\alpha = 1.2$, $\beta = 0.2$, $\kappa_2 = 1, 5, 10$, and one realization. In Fig. 2, $T = 1800$.

In Fig. 3a and b, $\omega = 0.00008, 0.00017, 0.00024, 0.00033, 0.0005, 0.009, 0.002,$ 0.0028, $0.0037, 0.005, 0.007, 0.013$; $\sigma = 0.05$; $\alpha = 1.1$; $\gamma = 0.02j$ for $j = 1, \cdots, 160$; and $\beta = (4-2\alpha)\gamma$. In Fig. 3c and d, $\omega = \sigma = 0$; $\alpha = 1.1$; $\gamma = 0.02j$ for $j = 1, \cdots, 160$; and $\beta = (4-2\alpha)\gamma$. In Fig. 3e and f, $\omega = 0.0003$; $\sigma = 0.05$; $\alpha = 0.001, 0.008, 0.2, 0.3, 0.4, 0.5, 0.6,$ $0.8, 1.0, 1.2, 1.5, 1.7$; $\gamma = 0.02j$ for $j = 1, \cdots, 160$; and $\beta = (4-2\alpha)\gamma$. In Fig. 3g and h, $\omega = \sigma = 0$; $\alpha = 0.001, 0.008, 0.2, 0.3, 0.4, 0.5, 0.6,$ $0.8, 1.0, 1.2, 1.5, 1.7$; $\gamma = 0.02j$ for $j = 1, \cdots, 160$; and $\beta = (4-2\alpha)\gamma$. In Fig. 3, $\kappa_1 = 1$; $\kappa_2 = 1$, 4; $n = 70$; $T = 2000$; and $X_{-i-1} = v_i$ for $i = 1, \cdots, 4$ where v_i is Gaussian white noise with $\sigma = 0.001$.

In Fig. 4a and b, $sd_{(i,im)}$ and $r_{5(i,im)}$ were computed using the original data X generated by equation (1) based on $\omega = 0.00008$; $\sigma = 0.01 + 0.02j$ for $j = 0, \cdots, 6$; $\alpha = 1.3$; $\beta = 0.1$; and $T = 10000$. In Fig. 4c–f, $sd_{(i,im)}$, sd_m,

$r_{5(i,im)}$ and r_{5m} for $i = 1, \cdots, 50$ and $m = 2, \cdots, 20$ were obtained using the original data X generated by equation (1) based on $\omega = 0.00008$; $\sigma = 0.01$; $\alpha = 1.3$; $\beta = 0.4$; and $T = 200000$.

In Fig. 5a and 5b, $\omega = 0.00008$; $\sigma = 0.001, 0.01, 0.03, 0.05, 0.07$; $\alpha = 1.1$, $\gamma = 0.02j$ for $j = 1, \cdots, 160$; and $\beta = (4 - 2\alpha)\gamma$. In Fig. 5c and 5d, $\omega = \sigma = 0$; $\alpha = 1.1$, $\gamma = 0.02j$ for $j = 1, \cdots, 160$; and $\beta = (4 - 2\alpha)\gamma$. In Fig. 5e and 5f $\omega = 0.00008$; $\sigma = 0.001$; $\beta = 0.001, 0.003, 0.005, 0.01, 0.04$; $\gamma = 0.02j$ for $j = 1, \cdots, 160$; and $\alpha = 2 - \frac{1}{2}\beta/\gamma$. In Fig. 5g and 5h $\omega = \sigma = 0$; $\beta = 0.001, 0.003, 0.005, 0.01, 0.04$; $\gamma = 0.02j$ for $j = 1, \cdots, 160$; and $\alpha = 2 - \frac{1}{2}\beta/\gamma$. In Fig. 5, $\kappa_1 = 1$; $m = 2$; $n = 50$; $T = 10000$; and $X_{-i-1} = v_i$ for $i = 1, \cdots, 4$ where v_i is Gaussian white noise with $\sigma = 0.001$.

In Fig. 6, $T = 50000$; $\omega = -4.475 \times 10^{-7}$; $\sigma = 0.0249$; for $\gamma = 0.0264$, $\alpha = 0.4973$ and $\beta = 0.0794$; for $\gamma = 1.25$, $\alpha = 1.6$ and $\beta = 1$.

In Fig. 7, $T = 1800$; $\omega_x = \omega_y = 0.0003$; $\sigma_x = \sigma_y = 0.05$; and $\beta_x = \beta_y = 0.1$ in each case; $(\alpha_{ix}, \alpha_{iy}) : (1.8, 0.2), (1.7, 0.3), (1.5, 0.4), (0.2.1.8), (0.3, 1.7), (0.4, 1.5), (1.8, 1.8), (0.8, 0.8), (0.2, 0.2)$, which corresponds to $(\eta_{1ix}, \eta_{1iy}) = (\omega_x/\alpha_{ix}, \omega_y/\alpha_{iy}) : (0.00017, 0.00150), (0.00018, 0.00100), (0.00020, 0.00075), (0.00150, 0.00017), (0.00100, 0.00018), (0.00075, 0.00020), (0.00017, 0.00017), (0.00038, 0.00038), (0.00150, 0.00150)$.

In Fig. 8, $T = 10000$; $\omega_x = \omega_y = 0.00008$; $\alpha_x = \alpha_y = 1.1$; $\beta_{ix} = \beta_{iy} = (4 - 2\alpha_x)\gamma_i$; $\gamma_i = 0.02i$ for $i = 1, \cdots, 49$; $\sigma_{jx} : 0.04, 0.03, 0.02, 0.01, 0.001$ for a given $\sigma_{jy} = 0.07$ in $\eta_{2ijx} < \eta_{2ijy}$, $\sigma_{jy} : 0.05, 0.04, 0.03, 0.02, 0.001$ for a given $\sigma_{jx} = 0.07$ in $\eta_{2ijx} > \eta_{2ijy}$, and $\sigma_{jx} = \sigma_{jy} : 0.07, 0.05, 0.03, 0.01, 0.001$ in $\eta_{2ijx} = \eta_{2ijy}$, namely, $(\sigma_{jx}, \sigma_{jy}) : (0.001, 0.07), (0.01, 0.07), (0.02, 0.07), (0.03, 0.07), (0.04, 0.07), (0.001, 0.001), (0.01, 0.01), (0.03, 0.03), (0.05, 0.05), (0.07, 0.07), (0.07, 0.001), (0.07, 0.02), (0.07, 0.03), (0.07, 0.04), (0.07, 0.05)$, which corresond to different amplitude indicators by $(\eta_{2ijx}, \eta_{2ijy}) = (\sigma_{jx}/\beta_{ix}, \sigma_{jy}/\beta_{ix})$.

In Fig. 9, using log(real data), but log(60/RR) (Fig. 9a, g, and h). In Fig. 9a, from top to bottom, 1th line: $\hat{\omega} = -0.000072, \hat{\sigma} = 0.01991, \hat{\alpha} = 0.9255$, and $\hat{\beta} = 0.1163$; 2th-line: $\hat{\omega} = 0.000022, \hat{\sigma} = 0.02062, \hat{\alpha} = 1.0257$, and $\hat{\beta} = 0.1118$; 3th-line: $\hat{\omega} = -0.000028, \hat{\sigma} = 0.01958, \hat{\alpha} = 0.8273$, and $\hat{\beta} = 0.1572$; and 4th-line: $\hat{\omega} = -0.000001, \hat{\sigma} = 0.01391, \hat{\alpha} = 1.1591$, and $\hat{\beta} = 0.1635$. In Fig. 9b, from top to bottom, 1th line: $\hat{\omega} = -0.000176, \hat{\sigma} = 0.02112, \hat{\alpha} = 1.1701$, and $\hat{\beta} = 0.0568$; 2th-line: $\hat{\omega} = -0.0000203, \hat{\sigma} = 0.0103, \hat{\alpha} = 1.3824$, and $\hat{\beta} = 0.1819$; 3th-line: $\hat{\omega} = 0.000000, \hat{\sigma} = 0.01691, \hat{\alpha} = 1.3837$, and $\hat{\beta} = 0.3129$; and 4th-line: $\hat{\omega} = -0.000001, \hat{\sigma} = 0.01396, \hat{\alpha} = 1.2100$, and $\hat{\beta} = 0.0708$. In Fig. 9c, $\hat{\omega} = -0.00004, \hat{\sigma} = 0.001944, \hat{\alpha} = 0.9656$, and $\hat{\beta} = 0.000085$ in 1971–1980 and $\hat{\omega} = -0.000001, \hat{\sigma} = 0.003090, \hat{\alpha} = 0.9257$, and $\hat{\beta} = 0.002456$ in 1981–2016. In Fig. 9d, $\hat{\omega} = 0.001737, \hat{\sigma} = 0.008713, \hat{\alpha} = 0.4642$, and $\hat{\beta} = 0.01578$ in 1927–1971 and $\hat{\omega} = 0.0008, \hat{\sigma} = 0.002922, \hat{\alpha} = 0.6442$, and $\hat{\beta} = 0.005196$ in 1972–2016. In Fig. 9e, $\hat{\omega} = 0.003393, \hat{\sigma} = 1.2781, \hat{\alpha} = 1.1160$, and $\hat{\beta} = 0.2853$ in 1700–1857 and $\hat{\omega} = 0.002284, \hat{\sigma} = 0.2451, \hat{\alpha} = 0.7081$, and $\hat{\beta} = 0.4626$ in 1858–2016. In Fig. 9f, $\hat{\omega} = 0.000165, \hat{\sigma} = 1.2781, \hat{\alpha} = 1.4412$, and $\hat{\beta} = 0.004782$ for monthly data and $\hat{\omega} = 0.001313$, $\hat{\sigma} = 0.6491, \hat{\alpha} = 1.2925$, and $\hat{\beta} = 0.5411$ for yearly data.

In Fig. 9g and h, $\hat{\omega} = -4.475 \times 10^{-7}$, $\hat{\sigma} = 0.02493$, $\hat{\alpha} = 0.4972$, and $\hat{\beta} = 0.0794$.

In the lines 126 and 127 below equation (4), the correlation coefficient between the wave indicators and the sd of the data generated by equation (1) were calculated based on the following parameters: for η_1: $\omega = 0.006 - 0.000015j$, $\sigma = 0.05$, $\alpha = 1.4 - 0.000521j$, and $\beta = 0.1$; for η_2: $\omega = 0.00019$, $\sigma = 0.000078125j$, $\alpha = 0.095$, $\beta = 0.1$, $j = 1, \cdots, 120$, $\kappa_2 = 1$ to 10, and $T = 2000$.

In the lines 185–197, when $\omega = 0.00600, 0.00065, 0.00042, 0.00030, 0.00022$; $\sigma = 0.05$; $\alpha = 1.40, 1.35, 1.30,$ 1.25, 1.2: we obtained the following estimations for DFA method: $H = 0.9000, 0.6700, 0.6757, 0.6815,$ and 0.6892 for $T = 1800$; $H = 0.8780, 0.6499, 0.6568, 0.6627,$ and 0.6682 for $T = 3000$; $H = 0.8362, 0.6080,$ $0.6168, 0.6226,$ and 0.6276 for $T = 8000$; for R/S method: $H = 1.0643, 0.9726,$ $0.9657, 0.9604,$ and 0.9545 for $T = 1800$; $H = 1.0642, 0.9729, 0.9667, 0.9600,$ and 0.9555 for $T = 3000$; $H = 1.0640, 0.9738, 0.9666, 0.9614,$ and 0.9552 for $T = 8000$.

3 Non-NLARI modeling

When $E\left|\dot{x}\right|$ is not small, we investigated whether Krylov–Bogolyubov averaging method could be used to deal with the self-regulating system. Self-regulation of natural systems is more frequently achieved by the attainment of equilibrium through negative feedback systems. Equation (1) can be derived by Zonglu He (2007). In the self-regulating system, the object or the system sustains the resistance force f, the restoration force g, and random force $\tilde{\epsilon}$. Newton's second law says that a net force results in a change of momentum: $F = d(m\dot{X})/dt$ of which $F = m\ddot{X}$ is just a special case only if mass m is constant. Without loss of generality, let $m = 1$. Then, $\ddot{X} = f + g + \tilde{\epsilon}$. The resistance function f should be the function of velocity $\dot{X}$ or the rate of change of position with delay τ_1, $f\left(\dot{X}\left(t - \tau_1\right)\right)$. The restoration function g should be the function of the deviation from equilibrium $\mu_t = E\left(X\left(t\right)\right)$ with delay τ_2, $g\left(X\left(t - \tau_2\right) - \mu_{t-\tau_2}\right)$. Let the mean $\omega = E\left(\tilde{\epsilon}\left(t\right)\right)$ and $\epsilon\left(t\right) = \tilde{\epsilon}\left(t\right) - \omega$ is a normal random variable. Thus, the self-regulating system can be described by

$$\ddot{X}\left(t\right) = \omega + f\left[\dot{X}\left(t - \tau_1\right)\right] + g\left[X\left(t - \tau_2\right) - \mu_{t-\tau_2}\right] + \epsilon\left(t\right) \qquad \text{(S4)}$$

Consider that the function h (f and g) must satisfy the conditions (i) $xh\left(x\right) < 0$ and $h\left(-x\right) = -h\left(-x\right)$ for $x \neq 0$ in order to characterize the nature of the resistance/restoration forces and (ii) a smooth integrable function to avoid an explosive solution. The conditions (i) implies that $g\left(0\right) = 0$, $\beta = -g'\left(0\right) > 0$, and $\alpha = -f'\left(0\right) > 0$. The function $f\left(x\right) = -\alpha x \exp\left(-x^2\right)$ satisfies the conditions for the resistance function. The first order Taylor polynomial for $g\left(x\right)$ near the point $x = 0$ is given by $g\left(x\right) \approx g\left(0\right) + g'\left(0\right)x = -\beta x$ when $E\left|x - E\left(x\right)\right|$ or $Var\left(x\right)$ is small. Therefore, equation (S4) without noise corresponds to the

deterministic system

$$\ddot{x}\left(t\right)=-\alpha\frac{\dot{x}\left(t-\tau_1\right)}{\exp\left(\dot{x}\left(t-\tau_1\right)^2\right)}-\beta x\left(t-\tau_2\right) \tag{S5}$$

When α is small (henceforth $\alpha\equiv\varepsilon$) and $\tau_1=\tau_2=0$, equation (S5) is an autonomous equation as the dynamic system $\ddot{x}=-\beta x-\alpha\dot{x}\exp\left(\dot{x}^2\right)$ and it can be written as

$$\begin{cases}\dot{x}=y\\ \dot{y}=-\beta x-\varepsilon y\exp\left(-y^2\right)\end{cases} \tag{S6}$$

To apply the Krylov–Bogolyubov averaging method to solve the approximate solution of equation (S6), the variable is transformed by $x=a\cos\left(\sqrt{\beta}t+\phi\right)$ and then $y=-a\sqrt{\beta}\sin\left(\sqrt{\beta}t+\phi\right)$. Let $\theta=\sqrt{\beta}t+\phi$. Equation (S6) can be rewritten as

$$\begin{cases}\dot{a}\cos\theta-a\dot{\phi}\sin\theta=0\\ -\dot{a}\sqrt{\beta}\sin\theta-a\sqrt{\beta}\dot{\phi}\cos\theta=-\varepsilon\left(-a\sqrt{\beta}\sin\theta\exp\left(-\left(a\sqrt{\beta}\sin\theta\right)^2\right)\right)\end{cases} \tag{S7}$$

which lead to

$$\begin{cases}\dot{a}=\frac{\varepsilon}{\sqrt{\beta}}\left(-a\sqrt{\beta}\sin\theta\exp\left(-\left(a\sqrt{\beta}\sin\theta\right)^2\right)\right)\sin\theta\\ \dot{\phi}=\frac{\varepsilon}{a\sqrt{\beta}}\left(-a\sqrt{\beta}\sin\theta\exp\left(-\left(a\sqrt{\beta}\sin\theta\right)^2\right)\right)\cos\theta\end{cases} \tag{S8}$$

Using the Krylov–Bogolyubov averaging method where $dt=\left(1/\sqrt{\beta}\right)d\theta$ yielded

$$\begin{cases}\dot{a}=\frac{\sqrt{\beta}}{2\pi}\int\limits_0^{2\pi/\sqrt{\beta}}\dot{a}dt=\frac{\varepsilon}{2\pi\sqrt{\beta}}\int\limits_0^{2\pi}\left(-a\sqrt{\beta}\sin\theta\exp\left(-\left(a\sqrt{\beta}\sin\theta\right)^2\right)\right)\sin\theta d\theta\\ \dot{\phi}=\frac{\sqrt{\beta}}{2\pi}\int\limits_0^{2\pi/\sqrt{\beta}}\dot{\phi}dt=\frac{\varepsilon}{2\pi a\sqrt{\beta}}\int\limits_0^{2\pi}\left(-a\sqrt{\beta}\sin\theta\exp\left(-\left(a\sqrt{\beta}\sin\theta\right)^2\right)\right)\cos\theta d\theta\end{cases} \tag{S9}$$

which leads to

$$\begin{cases}\dot{a}=-\frac{\varepsilon a}{2\pi}\int\limits_0^{2\pi}\sin^2\theta\exp\left(-a^2\beta\sin^2\theta\right)d\theta\\ \phi=\phi_0\end{cases} \tag{S10}$$

However, it is difficult to obtain the analytic solution of a in the above equation.

On the other hand, equation (S5) can be discretized into as follows:

$$X_t=\omega+2X_{t-1}-X_{t-2}-\beta X_{t-\kappa_2}+\beta\mu_{t-\kappa_2}-\varepsilon\frac{X_{t-\kappa_1}-X_{t-\kappa_1-1}}{e^{(X_{t-\kappa_1}-X_{t-\kappa_1-1})^2}}+\varepsilon_t \tag{S11}$$

where $\kappa_1=[\tau_1]$ and $\kappa_2=[\tau_2]$. From equation (S11), we have $\mu_t-2\mu_{t-1}+\mu_{t-2}=\omega+O\left(\varepsilon\right)$, which leads to $\mu_t=\omega t^2+\Delta$ where $\Delta\ll t^2$. When $\kappa_2=1$,

equation (S11) can be approximately expressed by $X_t - (2-\beta) X_{t-1} + X_{t-2} = \omega \left(1+\beta t^2\right)$. It can be proven that for $\beta \leq 4$, $|\lambda_i| = 1$; and for $\beta > 4$, $|\lambda_1| < 1$ and $|\lambda_2| > 1$, which implies that $|\lambda_2| \geq 1$. Let λ_i $(i = 1, 2)$ be the solutions of $\lambda^2 - (2-\beta)\lambda + 1 = 0$. Then,

$$\begin{aligned} |X_t| = \left|\tfrac{\omega\left(1+\beta t^2\right)}{(1-\lambda_1 L)(1-\lambda_2 L)}\right| &= \left|\omega\left(1+\beta t^2\right) \textstyle\sum_{i=1}^{t} \sum_{j=0}^{i-1} \lambda_2^i \lambda_1^j\right| \\ &\geq |\omega\lambda_2| \left(1+\beta t^2\right) \\ &\geq |\omega| \left(1+\beta t^2\right) \end{aligned} \quad \text{(S12)}$$

We can similarly prove that $|\lambda_i| > 1$ for $\kappa_2 = 2$. Consequently, we face the three difficulties in approximately solving equation (S11): (i) a small resistance coefficient ($\alpha = \varepsilon$) leads to the divergence of the self-regulating process; (ii) the original function of an odd integrable resistance function is not analytic; and (iii) the resistive/restorative delays will make this problem more complex. For these reasons, we consider the following model to explore the fractal behavior of nonlinear dynamics:

$$X_t = \omega + 2X_{t-1} - X_{t-2} - \alpha \frac{X_{t-\kappa_1} - X_{t-\kappa_1-1}}{e^{(X_{t-\kappa_1} - X_{t-\kappa_1-1})^2}} - \beta \frac{X_{t-\kappa_2} - \mu_{t-\kappa_2}}{e^{\left(X_{t-\kappa_2} - \mu_{t-\kappa_2}\right)^2}} + \varepsilon_t \quad \text{(S13)}$$

End#
